# Supramolecular motifs in dynamic covalent PEG-hemiaminal organogels

**DOI:** 10.1038/ncomms8417

**Published:** 2015-07-15

**Authors:** Courtney H. Fox, Gijs M. ter Hurrne, Rudy J. Wojtecki, Gavin O. Jones, Hans W. Horn, E. W. Meijer, Curtis W. Frank, James L. Hedrick, Jeannette M. García

**Affiliations:** 1 Department of Chemical Engineering, Stanford University, 443 via Ortega, Stanford, California 94305, USA; 2 Eindhoven University of Technology, Post Office Box 513, Eindhoven 5600 MB, The Netherlands; 3 IBM Almaden Research Center, 650 Harry Road, San Jose, California 95120, USA

## Abstract

Dynamic covalent materials are stable materials that possess reversible behaviour triggered by stimuli such as light, redox conditions or temperature; whereas supramolecular crosslinks depend on the equilibrium constant and relative concentrations of crosslinks as a function of temperature. The combination of these two reversible chemistries can allow access to materials with unique properties. Here, we show that this combination of dynamic covalent and supramolecular chemistry can be used to prepare organogels comprising distinct networks. Two materials containing hemiaminal crosslink junctions were synthesized; one material is comprised of dynamic covalent junctions and the other contains hydrogen-bonding bis-hemiaminal moieties. Under specific network synthesis conditions, these materials exhibited self-healing behaviour. This work reports on both the molecular-level detail of hemiaminal crosslink junction formation as well as the macroscopic behaviour of hemiaminal dynamic covalent network (HDCN) elastomeric organogels. These materials have potential applications as elastomeric components in printable materials, cargo carriers and adhesives.

## Abstract (web summary)

Control of supramolecular chemistry and dynamic covalent crosslinking are ideal ways to generate soft materials with desirable mechanical behaviours. Here, the authors report on practical and computational methods used to elucidate the structural properties of PEG-hemiaminal organogel networks.

## Graphical abstract

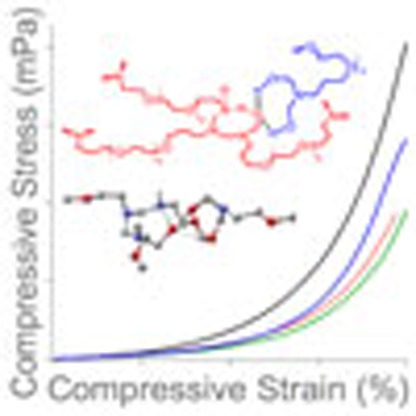

Organogels containing dynamic covalent crosslink junctions are a unique class of viscoelastic materials that can undergo network rearrangement, displaying a range of material properties including shape memory, modulated fluorescence and reworkability^1^,^2^,^3^,^4^. The repertoire of crosslinking chemistry has been expanded to include transient linkages through the use of dynamic bonds (both dynamic covalent bonds and supramolecular interactions). Dynamic covalent bonds are stable covalent bonds possessing dynamic behaviour that can be triggered by specific stimuli (for example, light, redox, temperature). Dynamic covalent crosslink junctions include disulfide bridges^5^, Diels-Alder linkages^4^,^6^ and TEMPO-functionalized radical groups^3^. Dynamic covalent crosslink junctions are stable until their reversible dynamic behaviour is triggered, initiating network rearrangement. A specific window exists for each dynamic bond in which the equilibrium can be manipulated to affect the disruption or formation of crosslinks.

On the other hand, supramolecular crosslinks depend on the equilibrium constant and relative concentrations of crosslinks as a function of temperature. Examples of supramolecular motifs include metal–ligand interactions^7^,^8^,^9^,^10^,^11^,^12^,^13^, inclusion complexes^14^,^15^, hydrogen-bonding groups^16^,^17^,^18^ and kinetically trapped species^19^. The combination of both physical crosslinks and dynamic covalent crosslinks enable the material to relax under load while still maintaining covalent stability^4^,^6^,^19^,^20^,^21^,^22^,^23^,^24^,^25^,^26^,^27^,^28^,^29^. The use of an organic solvent to form organogel materials permits the incorporation of water-insoluble compounds into a gel scaffold and affects the swelling behaviour of networks^1^,^2^,^30^.

Leibler and colleagues^28^ developed a thermoreversible rubbery elastomer through supramolecular assembly that exhibits rehealable behaviour^14^. Using Diels-Alder dynamic covalent bonds, Wudl and co-workers^21^ prepared strong highly crosslinked materials that are also thermally reversible^4^. The dynamic bonds formed in these materials produce crosslink junctions that can rearrange, which imparts self-healing or thermal reversibility to polymer networks.

Networks comprising solely irreversible covalent crosslink junctions are passive structures that do not reform following a mechanical failure. Recent advances in materials science have focused on developing rearrangeable networks and crosslink junctions; however, an inherent problem with continuously reversible (supramolecular) systems is that they suffer from creep due to thermal fluctuations of non-covalent linkages, which eventually leads to material failure. The deleterious effects of creep can be mitigated by the introduction of covalent crosslinks.

We previously reported a polymer synthetic scheme that enables the formation of kinetically trapped hemiaminal dynamic covalent crosslink junctions^31^. In this work, we explore and manipulate the equilibria involved in HDCN-forming reactions to trap reactive hemiaminal intermediates at covalent crosslink junctions; HDCN crosslinks can be combined with supramolecular motifs to access properties that are otherwise difficult to achieve using only dynamic covalent bonds^31^. The resulting hemiaminal crosslink junction is reversible with excess water; we observed that these materials have a strong affinity for coordinating solvents, such as *N*-methylpyrrolidone (NMP). Other aprotic, polar coordinating solvents such as dimethyl sulfoxide (DMSO), N-cyclohexyl-2-pyrrolidone (CHP) and dimethylformamide (DMF) are also viable for forming gels. Moreover, we established through computation that the coordinating solvent remained tightly bound and stabilized the hemiaminal intermediates (Fig. 1)^31^,^32^.

Herein, we report the formation of two distinct organogels: one that is a dynamic covalent network, and one that possesses both dynamic covalent and supramolecular bonding motifs. Detailed model studies (computational investigations and nuclear magnetic resonance (NMR) analysis) of the crosslinking reactions showed the effect of temperature and stoichiometry in the number of different products and types of species produced. Mechanical property measurements on bulk materials supported the insights concerning the chemical nature of HDCN crosslink junctions gained from model studies.

## Results

### Characterization of H/H and H/L organogels

**Figure 1:**
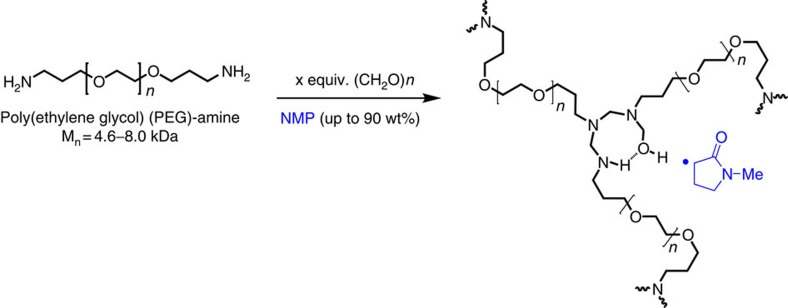
Synthesis of hemiaminal PEG Organogels. PEG-based HDCN organogels containing up to 90 wt. % N-methyl pyrrolidone (NMP).

Amine-terminated PEG oligomers react with paraformaldehyde in NMP to form PEG-HDCNs (Fig. 1). We carried out HDCN-forming reactions at 50 and 110 °C; the lower temperature (50 °C) is the minimum temperature for paraformaldehyde (PF) cracking to occur, while the higher reaction temperature (110 °C) is the upper limit for HDCN formation before network degradation at 120 °C. Detailed information regarding synthetic techniques used to produce HDCNs and model compounds is provided in Supplementary Tables 1–3. Four specific reaction conditions were used to synthesize PEG-HDCN organogels. We will use the following notation to describe these reaction conditions: H/H, H/L, L/H, L/L for HDCNs synthesized under the following respective conditions: 110 °C/4.4 equiv. paraformaldehyde (PF, equivalents relative to PEG-diamine), 110 °C/2.5 equiv. PF, 50 °C/4.4 equiv. PF, 50 °C/2.5 equiv. PF. We qualitatively observed that H/H gels self-heal at room temperature, whereas H/L, L/H and L/L gels exhibit elastomeric behaviour; materials with markedly different mechanical properties are produced at different reaction temperatures and paraformaldehyde ratios. A detailed description of this qualitative analysis is presented in Supplementary Fig. 1.

### NMR characterization

**Figure 2:**
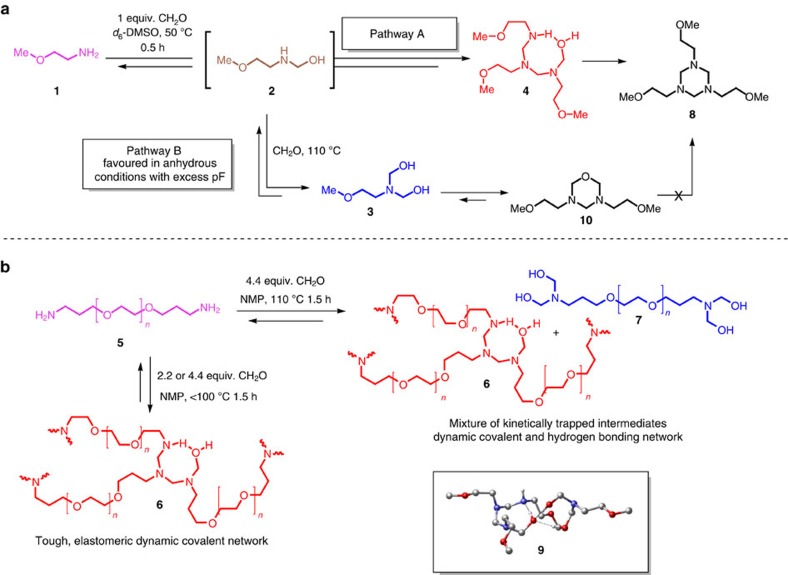
Equilibria for model compound and PEG HDCNs. (**a**) Model system based on soluble 2-methoxyethylamine (**1**) shows formation of different kinetic products based on reaction conditions determined by ^1^H, ^13^C, and 2D NMR analysis. Structures corresponding to hexahydrotriazine **8** and oxadiazinane **10** represent thermodynamically favored species not observed in PEG HDCNs. (**b**) The formation of kinetically trapped species in HDCNs and hydrogen bonding in HDCN gels (**9**, inset) which leads to observed self-healing behavior in gels synthesized at high temperature and 2.2 equivalents of paraformaldehyde relative to amine end groups.

The synthesis of hemiaminal crosslink junctions was studied using NMR of a solvent-soluble model reaction on a compound chosen for its electronic and steric similarities to PEG (2-methoxyethylamine, **1**). PEG-diamine is capable of forming a network after one amine end group reacts; however, this limits the mobility of unreacted amine end groups present in the gel. The mobility of the dissolved model compound is not limited since monofunctional amines do not form a percolated gel network. ^1^H and ^13^C NMR analysis showed formation of different kinetic products (Fig. 2a) depending on reaction temperatures (room temperature and 110 °C) and paraformaldehyde content (1.1 or 2.2 equivalents relative to amine end groups). NMR spectra for the model compound study used to develop Fig. 2 are presented in Supplementary Figs 2–12. The reactants and products of both the model compound-forming reaction and the PEG-HDCN-forming reaction are numbered in Fig. 2; we will directly refer to products discussed in this work using this numbering scheme.

Interestingly, structures corresponding to hexahydrotriazine (**8**) and *N,N,O*-heterocycle (oxadiazinane **10**) represent thermodynamically favoured structures not observed in PEG-HDCNs. Model reactions performed at room temperature showed the evolution of hemiaminal intermediates that ultimately react to either **8** or **10**, depending on reaction conditions. Only under conditions in which a stoichiometric excess of paraformaldehyde was present did we see evidence of the formation of **3**, which produces **10** through dehydration, imine formation and subsequent cyclization (Supplementary Fig. 16)^33^,^34^. We believe that the product associated with **3** is the kinetically trapped species that leads to H-bonding (see **9**, Fig. 2b inset and Supplementary Fig. 15) in the PEG network.

Model compound NMR studies indicate that the role of water as a catalyst for the intermolecular amine addition to imine and as proton shuttle for pathway A (Fig. 2a) is critical. For example, **4** was formed from the reaction of **1** with paraformaldehyde and **8** was observed within 40 min at room temperature when water was present (pathway A, kinetics determined by ^13^C NMR analysis, see Supplementary Fig. 12). When molecular sieves were added and anhydrous solvent and reagents were used, pathway A was drastically slowed and signals corresponding to intermediate hemiaminals were still observed even after 1 hour by ^1^H NMR; no hexahydrotriazine (**8**) was observed by NMR within this time frame (Supplementary Fig. 7). The thermodynamic product of pathway B (**10**) along with hexahydrotriazine (**8**) in a 1:2 ratio was observed after 7 h reaction time when 2.2 equivalents of paraformaldehyde to amine were used (Supplementary Fig. 8).

### Computational investigations

**Figure 3:**
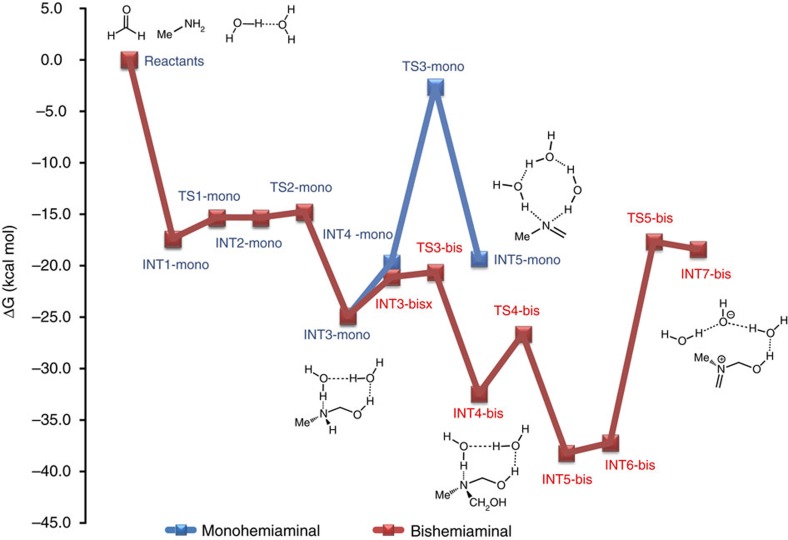
Free energy profile for the formation of 3 in excess paraformaldehyde. Mechanism and free energies for the reaction of MeNH_2_ with PF to form mono-hemiaminals, bishemiaminals and products formed after water loss.

Computational investigations were performed on methylamine, a computationally inexpensive model for PEG-diamine; these model reactions predicted very different chemical pathways depending on the reaction temperature and paraformaldehyde stoichiometry. The results of computational studies shown in Fig. 3 demonstrate that mono-hemiaminal formation is rapid, with a free energy barrier of only 3 kcal mol^−1^. After the hemiaminal is formed, it can either react with another molecule of PF, or form methylimine after water elimination. The computational methodology is provided in the Supplementary Information.

Previous studies suggest that the reaction of the mono-hemiaminal with formaldehyde is rapid with a free energy barrier of only 5 kcal mol^−1^, while water loss is much slower with a free energy barrier of 22 kcal mol^−1^ (refs 31, 32). The barrier for water loss from the bis-hemiaminal, 21 kcal mol^−1^, is almost equal to the barrier for water loss from the mono-hemiaminal, which is surprising given that an ion pair is formed after water loss. However, this can be rationalized by examining the transition state for water loss, which shows that the second hydroxyl group forms a hydrogen bond with one of the cocatalytic water molecules stabilizing the transition state. Note, however, that the ion pair formed after water loss from the bis-hemiaminal is expected to be short-lived. Consequently, our calculations predict that the bis-hemiaminal is preferentially formed in the presence of two equivalents of PF or greater. The bis-hemiaminal also expels water at a slightly faster rate and could lead to the formation of the *N,N,O*-heterocycle after further addition of amine and PF followed by ring-closure. We do not believe that HT is directly formed from *N,N,O*-heterocycle at high temperature. Instead, bis-hemiaminals and *N,N,O*-heterocycles formed during the reaction revert to the mono-hemiaminal, which in turn loses water and eventually forms hemiaminal trimer crosslink junctions by reacting with other amines present in the reaction.

The formation of hemiaminal trimer crosslink junctions produces either hemiaminal networks (**6**) or a mixture of hemiaminal and bis-hemiaminal crosslink junctions, **6** and **7** (Fig. 2b). The presence of both **6** and **7** forms a network that possess both dynamic covalent (**6**) and supramolecular (**7**) crosslink junctions. We anticipate that the dynamic covalent and supramolecular character of hemiaminal crosslink junctions will manifest in mechanical properties of HDCNs.

Model compound NMR and computational investigations focused on HDCN crosslink junction formation have shown that hemiaminal and bis-hemiaminal are formed. However, these model studies are limited and fail to capture the specifics of gel formation. A significant difference between the small molecule model system and PEG-HDCNs is that gelation precludes the formation of thermodynamic products shown in Fig. 2a (**8** and **10**), and, depending on stoichiometry, either the mono-hemiaminal or bis-hemiaminal is favoured.

In ^1^H NMR studies on the PEG-diamine network, we observed the formation of reaction temperature-dependent and paraformaldehyde concentration-dependent species (Supplementary Fig. 2). In some cases, these species were transient, while others persist over the course of the reaction. At 22 °C, we observed the appearance of a signal at ∼*δ*=2.87 p.p.m. and at ∼*δ*=3.13 p.p.m., attributed to hydrogen-bonded NMP trapped in the gel network. Transient signals corresponding to imine protons at ∼*δ*=7.00 p.p.m. and ∼*δ*=7.20 p.p.m., as well as unreacted paraformaldehyde, indicated a slow reaction to crosslinked products. ^1^H NMR of solutions reacted at higher temperatures lack the downfield signals corresponding to imine; the large signal corresponding to NMP and ethylene glycol repeating units obscures the hemiaminal and bis-hemiaminal signals, if present. We also explored the process of network reversion by comparing ^1^H-NMR spectra of HDCNs dissolved in D_2_O to HDCN networks (before the gel point) dissolved in *d*_6_-DMSO. Interestingly, trace water promotes the formation of hemiaminal covalent linkages from monofunctional amines but excess water promotes the breakdown of HDCN gels (Supplementary Figs 4 and 5).

Model compound studies indicate that hemiaminal intermediates are formed over the course of the condensation reaction. As a consequence, we anticipated that hemiaminal crosslink junctions formed in PEG-HDCNs would exhibit interesting mechanical properties. Thus, we explored the macroscale behaviour of HDCN networks containing hemiaminal and bis-hemiaminal crosslink junctions by characterizing the mechanical properties of HDCNs using compressive mechanical testing and oscillatory rheology.

### Mechanical testing

**Figure 4:**
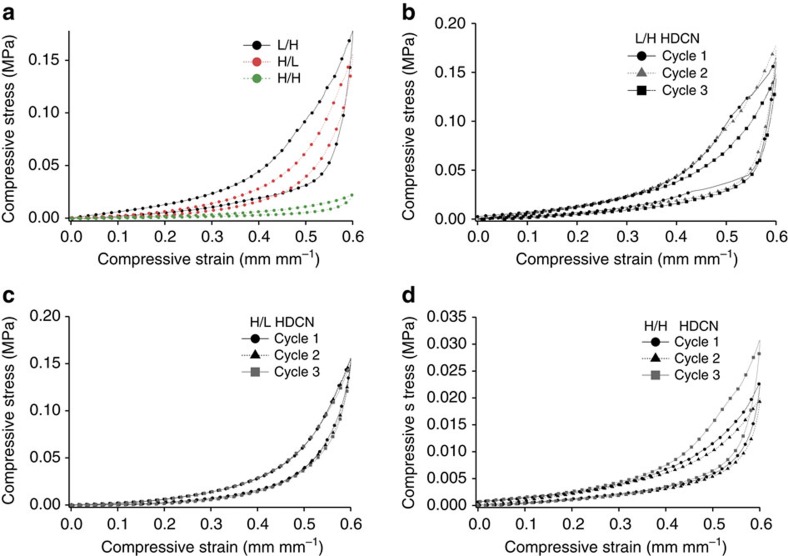
Mechanical testing of organogels prepared under different conditions. (**a**) Hysteresis curves for H/H, H/L, and L/H HDCNs. (**b**) Hysteresis curves for three load/unload cycles, L/H HDCN. (**c**) Hysteresis curves for three load/unload cycles, H/L HDCN. (**d**) Hysteresis curves for three load/unload cycles, H/H HDCN.

Figure 4 shows the hysteresis behaviour of HDCN organogels synthesized under specific reaction conditions. Dynamic mechanical analysis (DMA) results are presented in Fig. 6 and Supplementary Fig. 14. Images of stretched HDCN gels are provided in Supplementary Fig. 14. HDCN gels were linearly deformed at a rate of 0.1 (mm mm^−1^) per minute over a strain range within the elastic deformation regime (<60% strain). All HDCN gels exhibited elastomeric behaviour when strained <0.6 mm mm^−1^; the hysteresis curves indicate that no residual strain was present in the network after each test. All gels exhibited hysteresis behaviour, and the energy dissipated by the load–unload cycles was measured by calculating the area between the load–unload curves.

The mechanical properties of HDCN organogels are heavily dependent upon the reaction conditions used to produce these gels. H/L HDCNs have modulus values comparable to L/H gels, while the modulus of self-healing H/H organogels is an order of magnitude lower than the modulus of gels synthesized at lower temperatures. Energy dissipation during load–unload cycles (Table 1) can be attributed to molecular-level changes in the HDCN structure leading to deviations from Hookean behaviour^35^. For a Hookean elastomer, the modulus is expected to increase linearly with displacement with no associated energy loss. Energy dissipation upon deformation (Table 1) is attributed to deviations from ideal elastomer behaviour. Gels synthesized with stoichiometric paraformaldehyde equivalencies dissipate two to eight times as much energy in each cycle as those synthesized at a stoichiometric excess of paraformaldehyde quantities. L/H HDCNs dissipate twice the energy as H/L gels, yet the modulus values for these gels are similar. We attribute the difference in energy dissipation to molecular-level differences in HDCN crosslink junctions as a result of forming products **6** and **7** under different reaction conditions.

**Figure 5:**
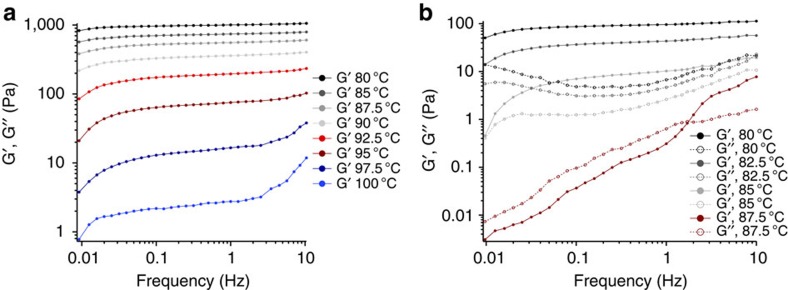
Rheology of H/H and H/L organogels. Dynamic oscillatory shear rheology of HDCNs cooled from 105 °C to 80 °C with (**a**) 2.0 equivalents of paraformaldehyde and (**b**) 4.4 equivalents of paraformaldehyde (Data from T>87.5 °C was noisy and was not included. G″ data for H/L HDCNs collected at T>87.5 °C were not included for clarity's sake. For this range, G′ was an order of magnitude greater than G″. Dynamic mechanical measurements were performed within the linear viscoelastic regime, γ=1%.).

**Figure 6:**
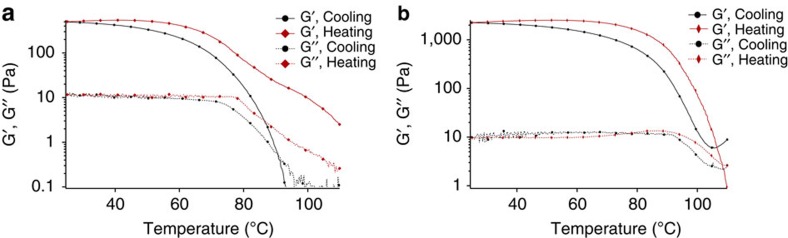
DMA Analysis of H/H and H/L Organogels. Temperature-dependent dynamic mechanical behavior of (**a**) H/H and (**b**) H/L HDCN organogels. Two transitions are evident in H/H HDCNs which we attribute to the breaking of hydrogen bonding and dynamic covalent bonds, respectively.

We characterized the temperature-dependent mechanical properties of H/L and H/H HDCNs by gradually cooling HDCNs from the reaction temperature (110 °C). A sol–gel transition was observed for H/H HDCNs near *T*=87.5 °C (G'∼G”∼ω^d^), while the storage modulus (G') of H/L HDCNs increased dramatically as the reaction mixture was cooled (Fig. 5)^36^. H/L HDCNs exhibit gelation (G'>G”) at *T*=100 °C. Both H/H and H/L HDCN organogels form an elastomeric material after the gel has cooled to 80 °C. The temperature-dependent increase in the storage modulus shown in Fig. 5 indicates that the reaction temperature governs the formation of hemiaminal trimer crosslink junctions. Heating an H/H elastomeric gel from 20 to 110 °C reverted HDCN precursors to a viscous liquid at higher temperatures but the storage and loss moduli values do not revert to the initial values of the storage and loss modulus (Fig. 6).

DMA conducted over the range of *T*=110 to 20 °C mimics the gradual cooling that leads to the formation of elastomeric H/H and H/L HDCNs. Temperature-dependent oscillatory frequency measurements presented in Fig. 5 show the changes in the storage and loss moduli of organogels above and below the boiling point of water. At temperatures below the boiling point of water, the storage modulus reaches a plateau, indicating that an elastomeric organogel has formed. After HDCN organogels were reheated to temperatures >100 °C, the network softened and eventually broke down to its solubilized liquid precursors, as indicated by a gradual decline in the storage modulus (Supplementary Fig. 13). The temperature-dependent increase in the storage modulus of both H/L and H/H HDCNs shown in Fig. 4 indicates that the crosslink junctions form at specific temperatures.

DMA of H/H and H/L gels show a remarkable difference in G' upon heating (Fig. 6). In the DMA of the H/H HDCN, two transitions are observed in the G' during heating. We attribute the first thermal transition to the breaking of hydrogen bonds in the network due to the presence of hydrogen-bonded hemiaminal dangling chain ends produced under these conditions (**7**). The second observable transition we attribute to breaking bonds at the dynamic, covalent trimer crosslink junctions (**6**). However, when H/L conditions were used, gels displayed one thermal transition, consistent with energy dissipation assigned solely related to the breaking of bonds in the trimer crosslinks.

**Figure 7:**
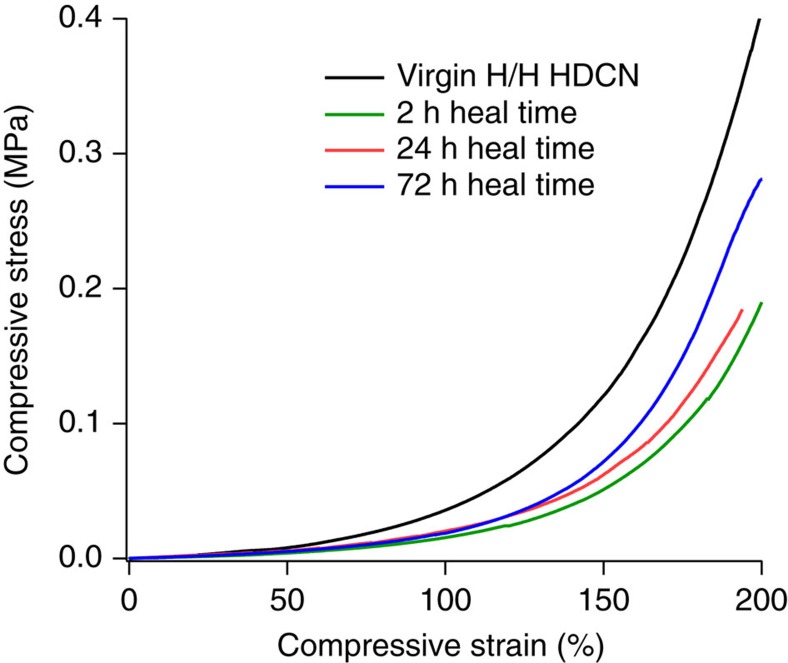
Mechanical analysis of self-healing behavior in H/H organogels. Self-healing H/H organogels regain a portion of the original compressive strength that increases after a given heal time at ambient temperature.

Self-healing behaviour was observed for gels synthesized at 110 °C with stoichiometric excess of paraformaldehyde. Figure 7 includes compressive stress–strain plots for unfractured H/H HDCNs and HDCNs fractured at an initial time (*t*=0) and permitted to heal for varied time periods. After 72 h (3 days), the strain-at-break had approached the strain-at-break value for the virgin material. In the elastic regime (<60% strain), the modulus values were comparable after only 2 h of healing time. The high extensibility of these networks after short healing periods indicates that H/H HDCNs are self-healing materials. Moreover, rheological characterizations of the gelation of HDCN networks indicate that the gel formation process is thermally reversible, confirming that HDCN-forming bonds are dynamic covalent. The temperature dependence of gel formation (Fig. 5) and the self-healing behaviour (Fig. 7) confirm that H/H networks exhibit both self-healing and temperature-dependent dynamic covalent character.

## Discussion

Density functional theory (DFT) calculations and experimental results indicate that the hemiaminal linkage exists in a dynamic equilibrium with dissolved formaldehyde and complexed water to form the organogel structure. DFT calculations also indicate that bound water plays a critical role in stabilizing the hemiaminal–NMP complex^31^,^32^. Results from computational investigations (Fig. 3) indicate that bound water drives the formation of hemiaminal cross-link junctions at lower temperatures. Only ∼3.0 mg of water is required to catalyse hemiaminal trimerization. Thus, it is plausible that sufficient water is present in conditions in which rigorous drying is not used to promote the formation of hemiaminal linkages.

We propose that a temperature-dependent equilibrium between NMP-soluble formaldehyde, water and hemiaminal covalent crosslink junctions creates a thermally reversible dynamic covalent gel that can be trapped under the right conditions. Gels synthesized with stoichiometric quantities of paraformaldehyde with respect to amine end groups form reversible hemiaminal linkages, while gels that are synthesized with two equivalents of paraformaldehyde exist as a thermoreversible dynamic system. The presence of thermally reversible covalent linkages as well as hydrogen-bonding interactions in the network was evidenced by mechanical testing of gels synthesized at high temperature with excess paraformaldehyde.

The self-healing nature of H/H organogels is attributed to the production of a mixture of **6** and **7**, which leads to the formation of hydrogen bonds between chain ends of **7** and crosslink junctions in **6** (Fig. 2 inset, **9**). This hydrogen-bonding motif sustains the self-healing behaviour observed in H/H organogels. We attribute the energy dissipation observed in hysteresis studies (*vide infra*, Fig. 4) to the formation and disruption of these hydrogen bonds within the network. Notably, **7** is an end-capped dangling chain that simultaneously serves as a network defect that promotes supramolecular versus covalent crosslinking. For gels synthesized at <100 °C, water catalyses polycondensation exclusively and quickly to form highly crosslinked **6** (gels typically form within ∼15 min of reaction); the resulting elastomeric gels formed at low temperatures did not display self-healing behaviour. In the absence of organic solvent, the compressive Young's modulus of ‘dried' lyophilized HDCNs was 36 MPa, indicating that modifying the solvent content of PEG-HDCNs provides another means of tuning the mechanical behaviour of HDCN materials.

The mechanical properties of HDCN networks are highly dependent on the reaction temperature and stoichiometry. We attribute the thermally reversible behaviour of gels synthesized at higher temperatures to the presence of water in the reaction mixture. As gels are cooled from 110 °C, water promotes the formation of hemiaminal trimer linkages at temperatures below the boiling point of water. The thermally reversible mechanical properties shown in Fig. 6 demonstrate that water remaining in the sealed system allows for the reconstitution of the original gel (that is, thermoreversibility).

The L/H gels had the same paraformaldehyde content as self-healing gels but did not exhibit self-healing behaviour at room temperature. The lack of self-healing supports the notion that trace water (which remains in the NMP solution at 50 °C) promotes the formation of **6** as predicted by DFT calculations^31^,^32^. Hemiaminal trimer crosslink junctions throughout the HDCN produce a densely crosslinked elastomeric network^37^,^38^,^39^. Compressive mechanical measurements performed on HDCNs demonstrate that these organogels behave as elastomers and do not lose solvent when force is applied, which is surprising given that the gels contain ∼90% solvent.

Model reactions carried out at high temperatures with excess paraformaldehyde show the formation of a mixture of possible species, including hemiaminal **2**, bis-hemiaminal **3** and hexahydrotriazine **8**. However, in the PEG system, hexahydrotriazine is not formed due to the limited mobility of PEG-diamine end groups. Hemiaminal and bis-hemiaminal species will hydrogen bond to one another (**9**), providing a rationale for the observed self-healing properties.

In summary, we have developed a thermoset, PEG-based organogel that exhibits dynamic covalent behaviour. The dynamic covalent behaviour of crosslink junctions led to unique temperature-dependent mechanical behaviour, resulting in the formation of a self-healing network at high temperatures, and a rubbery, strain-hardening elastomer at low temperatures. The unique self-healing behaviour is a result of the formation of network ‘defects' that we have attributed to the kinetically driven formation of hydrogen-bonding bis-hemiaminal species at elevated temperatures. We captured the formation of these kinetically driven products through NMR studies using a model compound. More broadly, we have presented a means for relating the molecular-level behaviour of network crosslink junctions to the structural properties of PEG organogels, which can lead to the development of other dynamic covalent elastomers.

## Methods

Paraformaldehyde, 2-methoxyethylamine and NMP were purchased from Sigma-Aldrich and used as received unless otherwise specified. The *d*_*6*_**-DMSO was purchased from Cambridge Isotope Labs and used as received unless otherwise specified. Poly(ethylene glycol) diol 4.6 kDa and 8.0 kDa were end-functionalized with primary amines using a previously developed protocol^40^. PEG-diamine was reacted with varying equivalents of paraformaldehyde in NMP to form crosslinked organogels, wherein the crosslink junctions are kinetically trapped hemiaminal linkages. The reaction temperature was controlled using a mineral oil bath at varying temperatures and allowed to reach thermal equilibrium before reaction (50, 80, 110 and 115 °C, typical times for oil bath to reach equilibrium ∼1 h). Reagents were dissolved in NMP by briefly heating and stirring the reaction mixtures, then incubating the gels in mineral oil baths for 90 min. After 90 min, the gels were removed from the oil bath and cooled to an ambient temperature (22 °C). Supplementary Fig. S1C describes the qualitative properties of HDCN gels synthesized at various temperatures and paraformaldehyde content. We have demonstrated (qualitatively) that HDCNs form self-healing networks when synthesized at high temperatures with >2 equivalents of paraformaldehyde per amine end group. Notably, when >4.4 equivalents of paraformaldehyde were reacted, no gel was formed, which is evidence of nearly complete end-capping of the diamine under these conditions.

## Additional information

**How to cite this article:** Fox, C.H. *et al*. Supramolecular motifs in dynamic covalent PEG-hemiaminal organogels. *Nat. Commun.* 6:7417 doi: 10.1038/ncomms8417 (2015).

## Supplementary Material

Supplementary Information Supplementary Figures 1-16, Supplementary Tables 1-3, Supplementary Discussion, Supplementary Methods and Supplementary References

Supplementary Data 1 Cartesian coordinates and energies for reactants, intermediates and transition structures in the reaction of methylamine with formaldehyde

## Figures and Tables

**Table 1:** Energy dissipated during compression cycles expressed in J mol^−1^ PEG. Moduli calculated using the ratio of stress to strain at 60% strain from the first hysteresis cycle.

**Cycle Number**	**L/H HDCN Modulus 280 kPa**	**H/L HDCN Modulus 250 kPa**	**H/H HDCN Modulus 41 kPa**
1	1,000	435	115
2	789	468	100
3	884	496	160
