# Supplementary material for: Supramolecular motifs in dynamic covalent PEG-hemiaminal organogels
Source: Nat Commun. 2015 Jul 15;6:7417. doi: 10.1038/ncomms8417 (PMC4518264; doi:10.1038/ncomms8417)
Supplement: Supplementary Information — Supplementary Figures 1-16, Supplementary Tables 1-3, Supplementary Discussion, Supplementary Methods and Supplementary References [file ncomms8417-s1.pdf]

Supplementary Figures

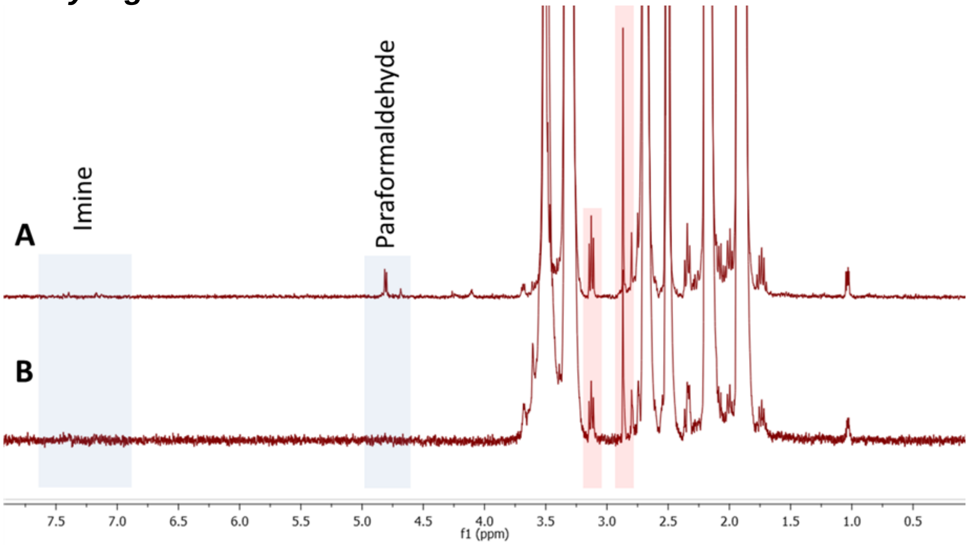

C

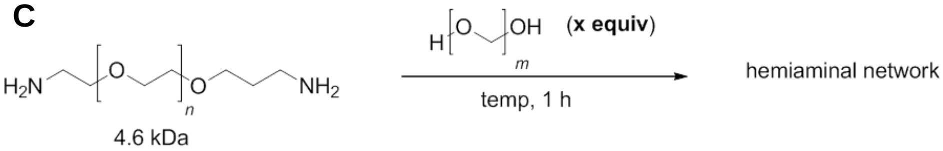

entry	equiv paraformaldehyde	temp (°C)	gel formed?	self healing?
1	2.5	22	Yes, after 24 h; opaque	Transient
2	2.5	50	Yes, within the hour; mostly clear	Transient
3	2.5	80	Yes, within the hour; mostly clear	Transient
4	2.5	115	Yes, after cooling to 22 °C; clear yellowish	Transient
5	4.4	115	Yes, after cooling to 22 °C; clear yellowish	Sustained
**6**	**4.4**	**110**	**Yes, after cooling to 22 °C; clear and colorless**	**Sustained**
7	8.8	110	NO	N/A

Figure S1. Characterization of PEG networks under various conditions. (**A**) ^{1}H-NMR of aliquot of mixture taken at 22 °C. Downfield shifted peaks of the related imine are visible at ~ δ 7.40 ppm (doublet) and ~ δ 7.14 ppm (doublet) as well as unreacted

paraformaldehyde at ~ δ 4.80 (multiplet). Also observed are signals at ~ δ 3.13 ppm (triplet) and ~ δ 2.87 ppm (singlet) corresponding to hydrogen-bonded NMP in the PEG HDCN. (**B**) Aliquot of sample after heating to 115 °C prior to gelation. No signal corresponding to imine is observed. (**C**) Optimization of conditions for self-healing behavior.

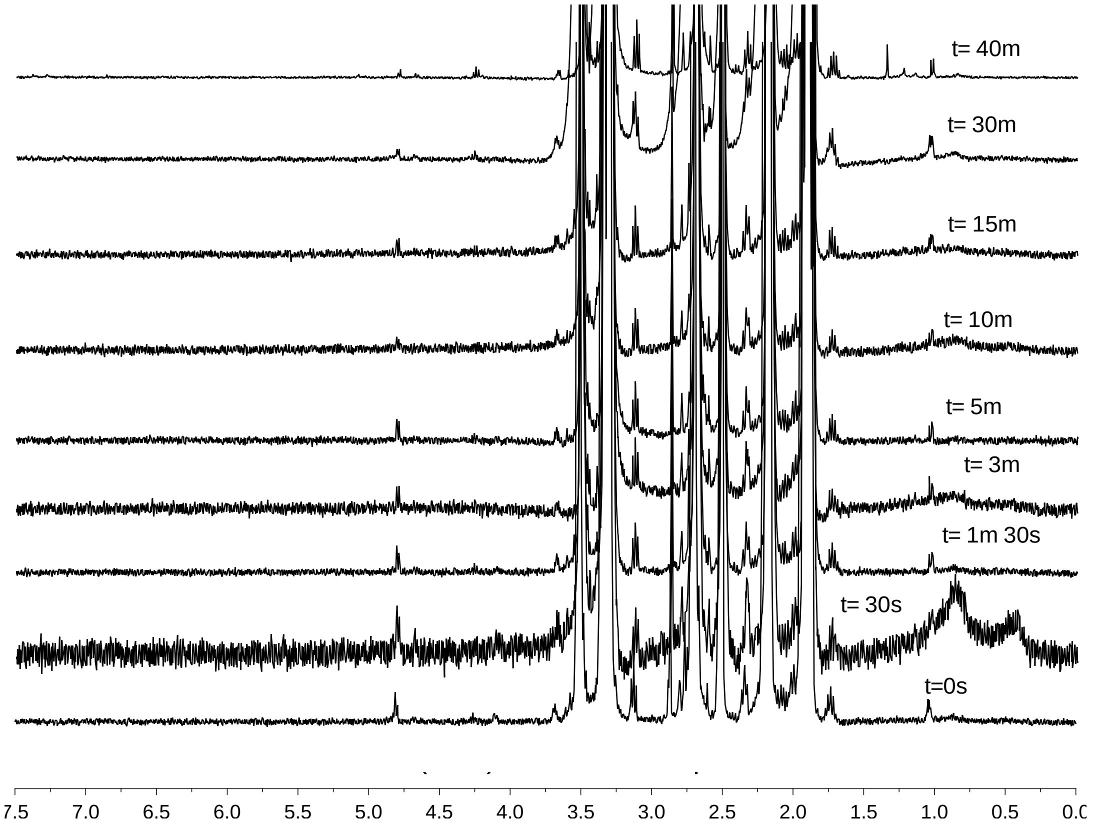

Figure S2. Kinetic NMR study of PEG diamine reacting in dilute conditons. ^{1}H NMR of aliquots of PEG-diamine and paraformaldehyde at 50 °C, sampled over for t< $t_{gelation}$. The signal observed at δ ~ 1.05 ppm is related paraformaldehyde after heating.

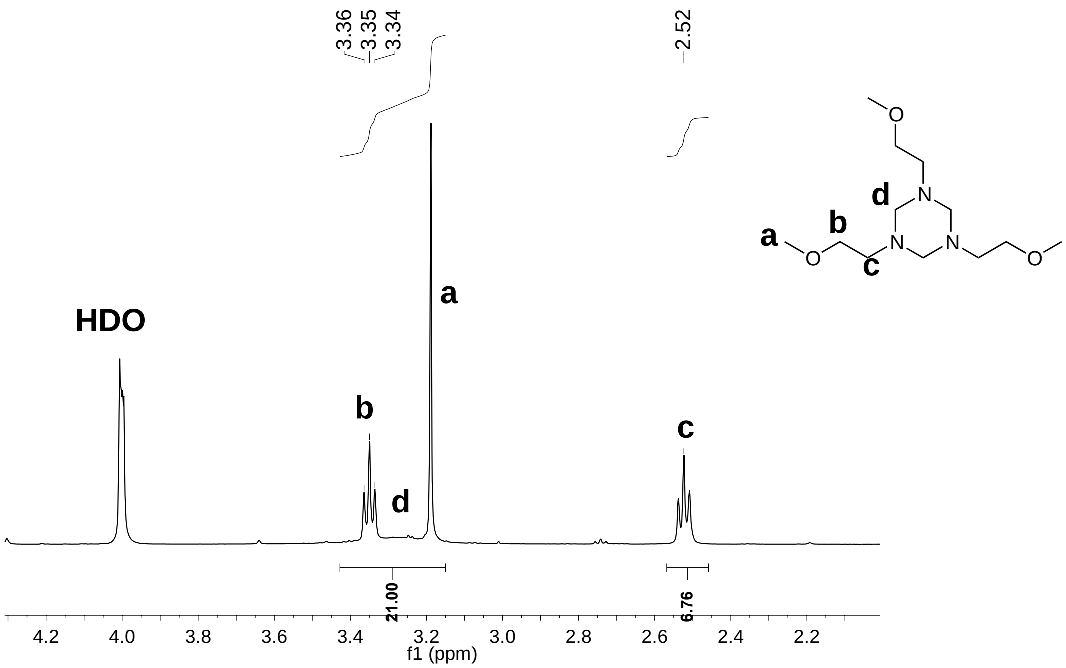

Figure S3. Model study of HT reaction with water. ^{1}H NMR scan of a 2-methoxyethylamine, 2.2 equiv. PF, 110 °C NMR-scale reaction to **8** after 22 hours soaking in D_2O. No change in the spectrum was observed within this timeframe, indicating stability of **8** to neutral, aqueous conditions.

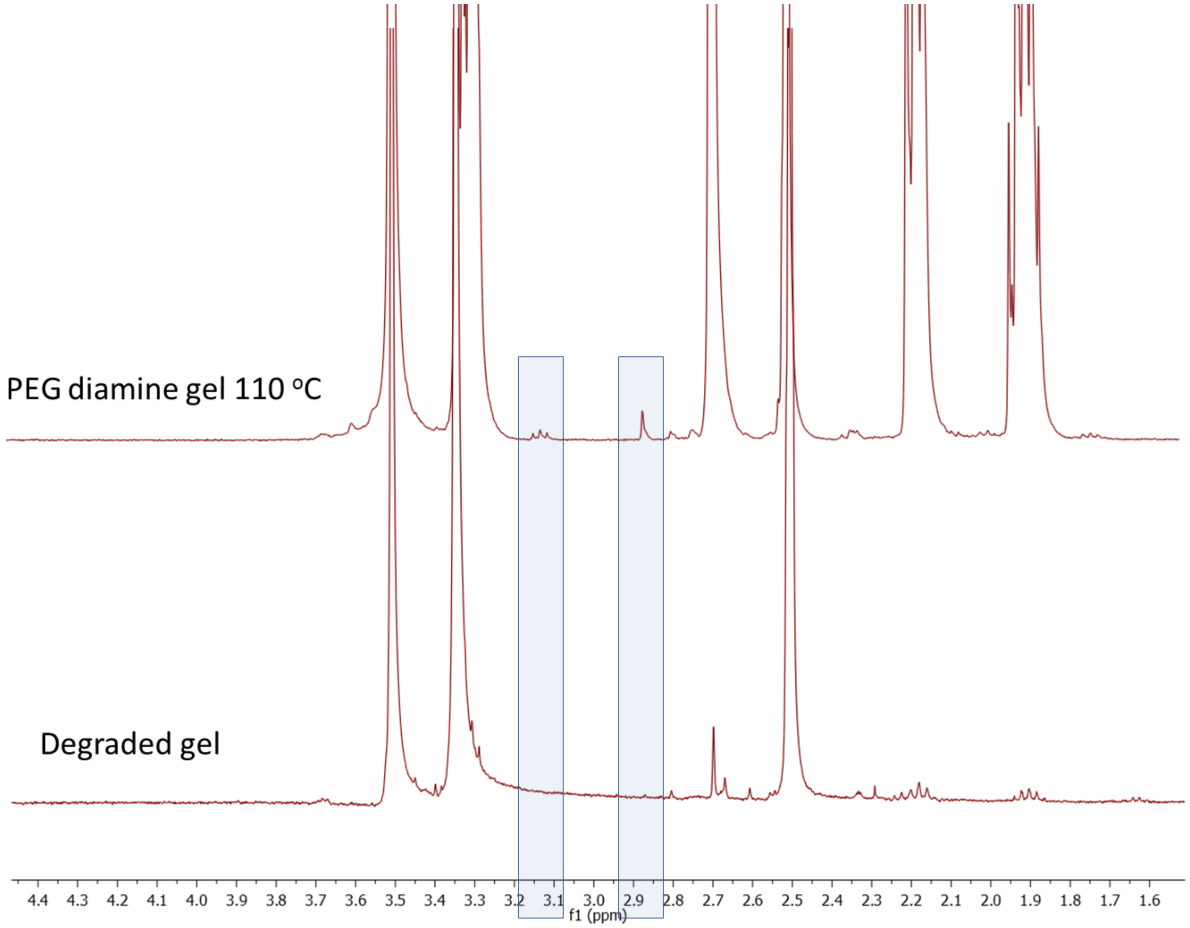

Figure S4. PEG diamine network before and after reversion in water. ^{1}H-NMR spectra in d_6-DMSO of (**A**) an aliquot of a HDCN forming reaction at T=110°C at $t < t_{gel}$ (t = 30 min.) and (**B**) a HDCN degraded for 4 h at 110°C in H_2O.

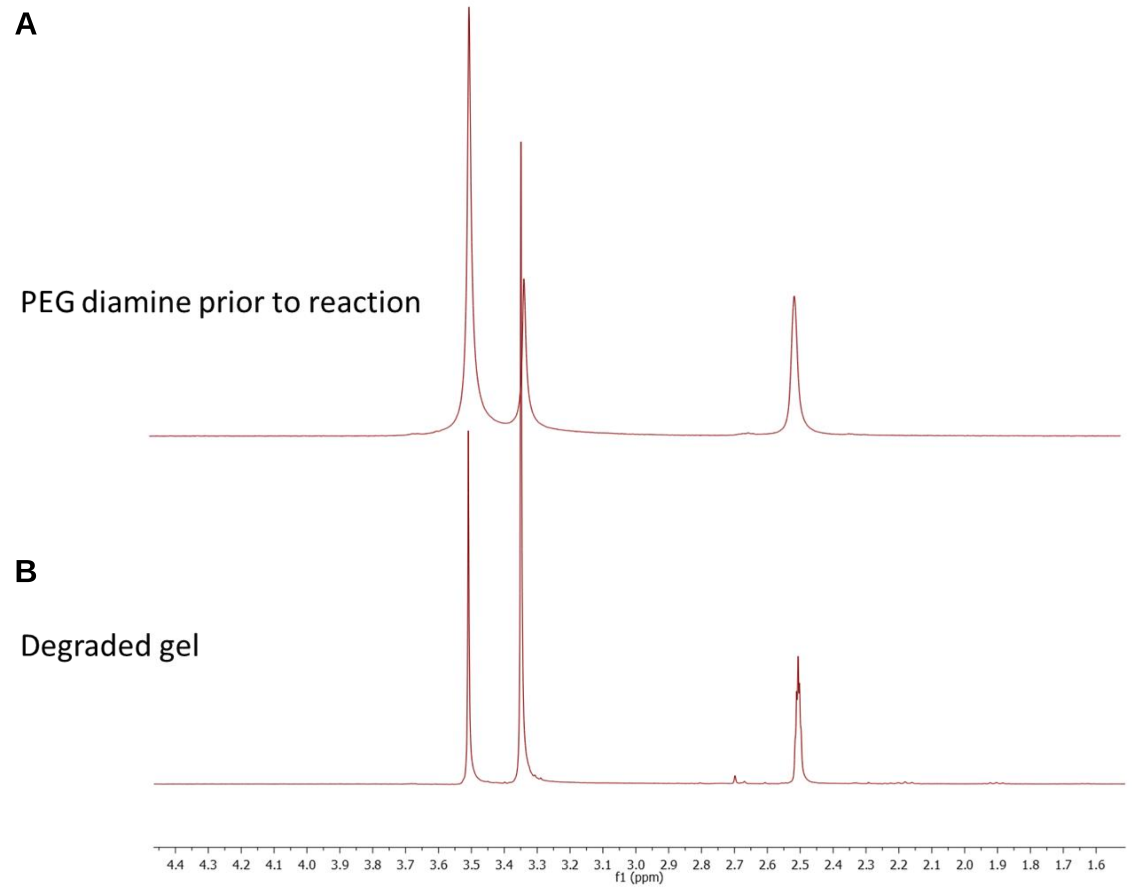

Figure S5. PEG diamine is recovered after water treatment of gel. (**A**) PEG diamine prior to reaction, then (**B**) after gel is degraded in water.

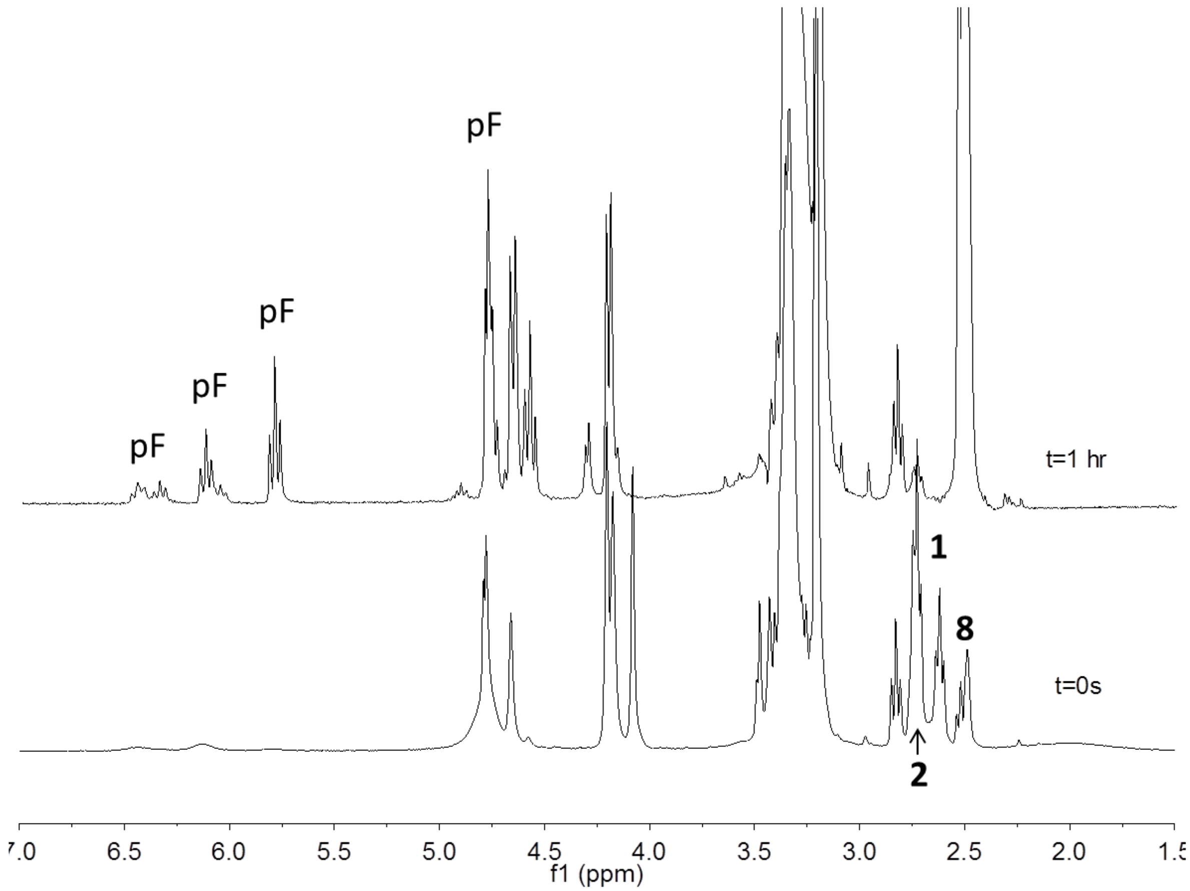

Figure S6. Hemiaminal intermediates are observed at room temperature over one hour. ^{1}H NMR of 2-methoxyethylamine (**1**), 2.2 equiv. PF, 20°C, 20 mg-scale reaction at t = 0 s and t = 1 h. Immediate conversion to a complex mixture of hemiaminal (**2**) and hexahydrotriazine (**3**) species are seen instantaneously, in contrast to the PEG system in which a smaller set of signals are observed and hexahydrotriazine is not formed.

A

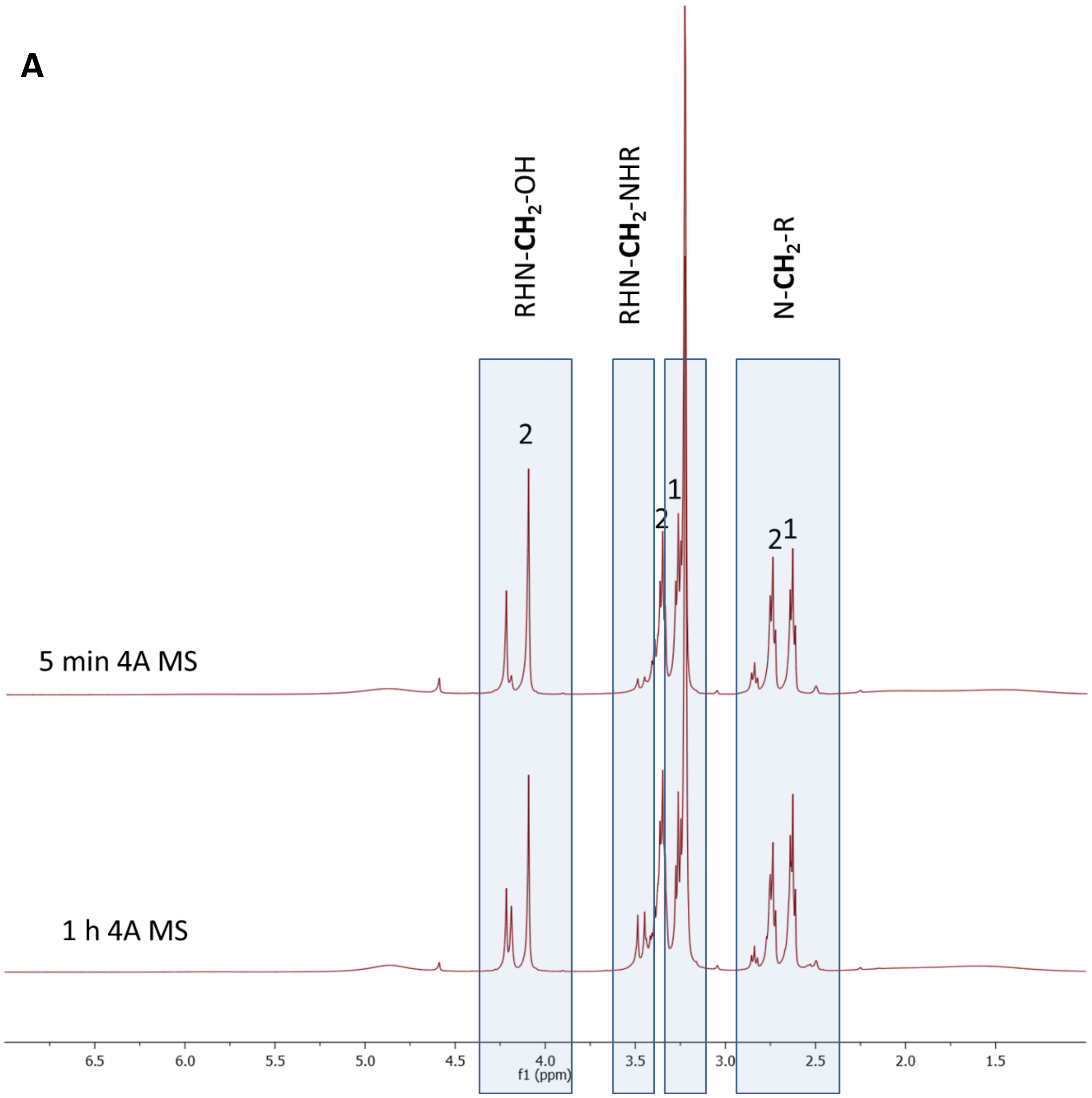
RHN-CH2-OH
RHN-CH2-NHR
N-CH2-R
2
1
2
2
1
5 min 4A MS
1 h 4A MS
6.5
6.0
5.5
5.0
4.5
4.0
f1 (ppm)
3.5
3.0
2.5
2.0
1.5

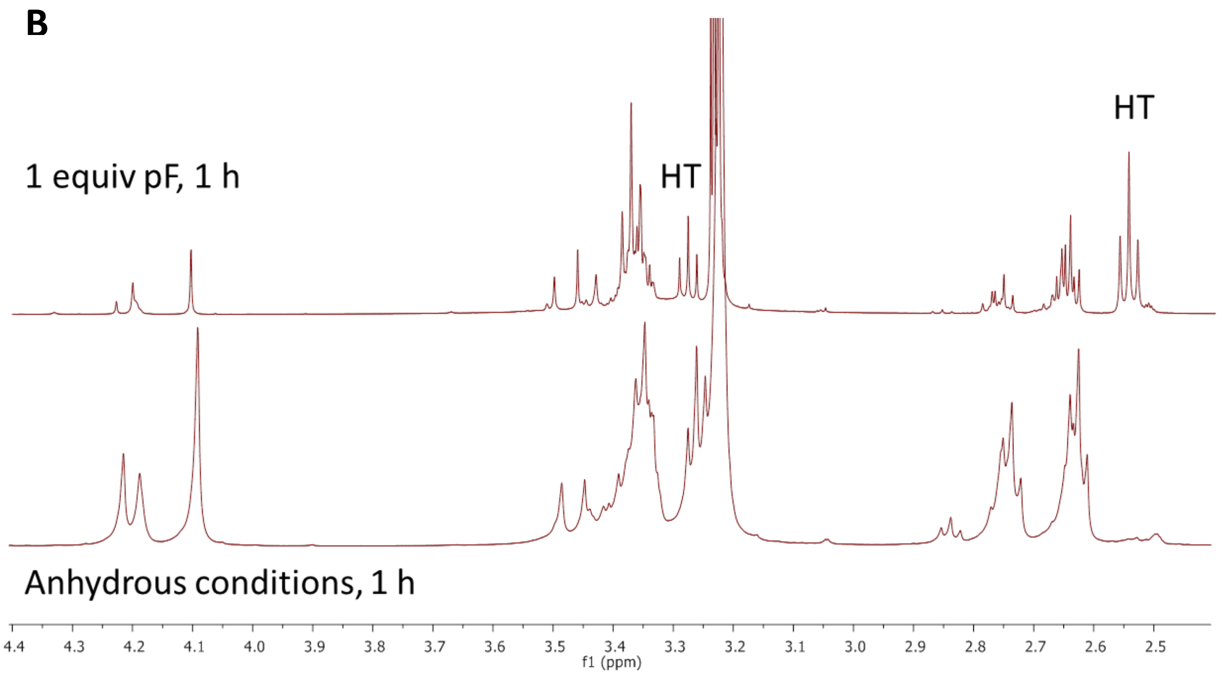

Figure S7. Effect of water on kinetics. (**A**) Kinetic ^{1}H NMR data for **1** reacting with 2.2 equivalents paraformaldehyde at room temperature in anhydrous d_6-DMSO and with dried **1**. (**B**) Reaction after 1 h at 22°C of **1** with 1.0 equiv. paraformaldehyde in "wet" conditions (top trace), compared to anhydrous conditions after 1 h (bottom trace). HT **8** is not observed after 1 h in anhydrous conditions.

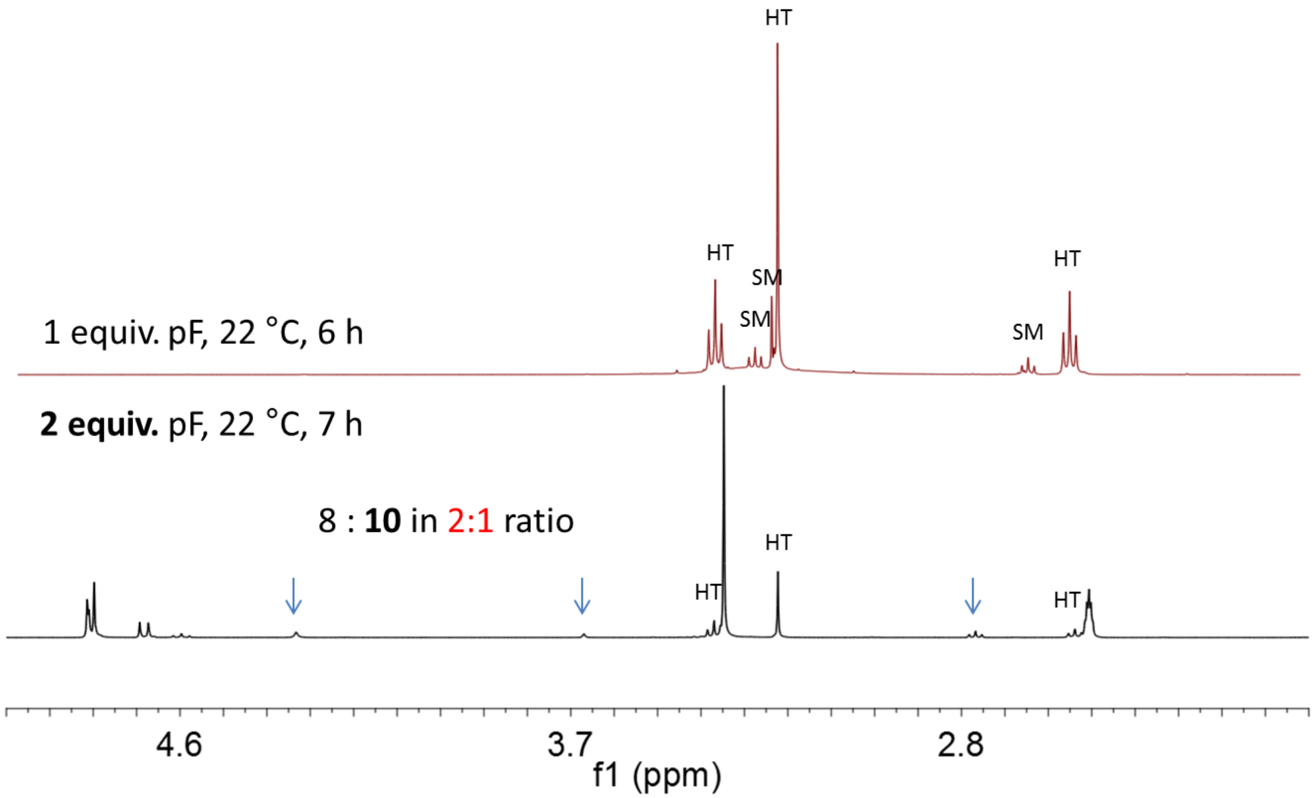

Figure S8. Thermodynamic products observed with stoichiometric excess of paraformaldehyde. Reaction at 22°C of **1** with 1.0 or 2.0 equiv. paraformaldehyde in "wet" conditions. A 2:1 mixture of **8**:**10** is observed with 2.0 equiv. paraformaldehyde (bottom trace), whereas with stoichiometric paraformaldehyde **10** is not observed (top trace).

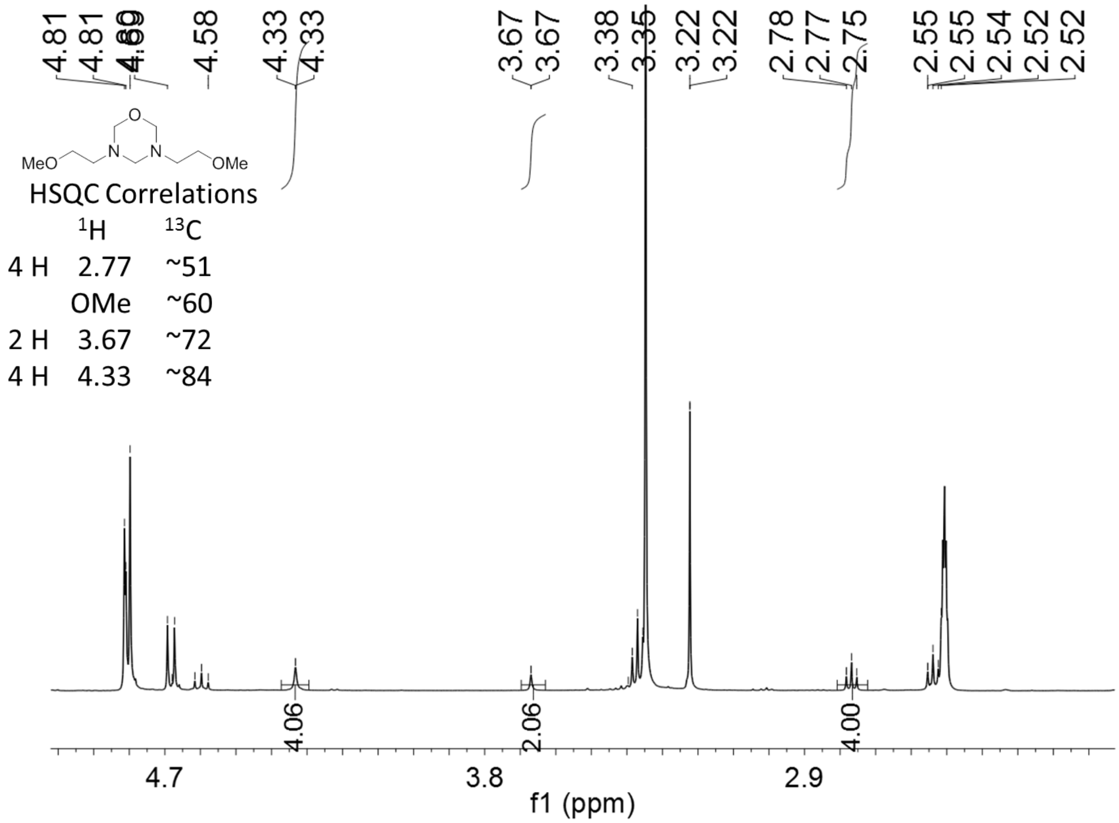

Figure S9. ^{1}H NMR characterization of oxadiazinane. ^{1}H NMR data corresponding to HSQC data below of **1** with paraformaldehyde at room temperature after 7 hours. Signals at ~ δ = 4.7 ppm, including the triplet observed at δ = 4.58 ppm correspond to paraformaldehyde in d_6-DMSO.

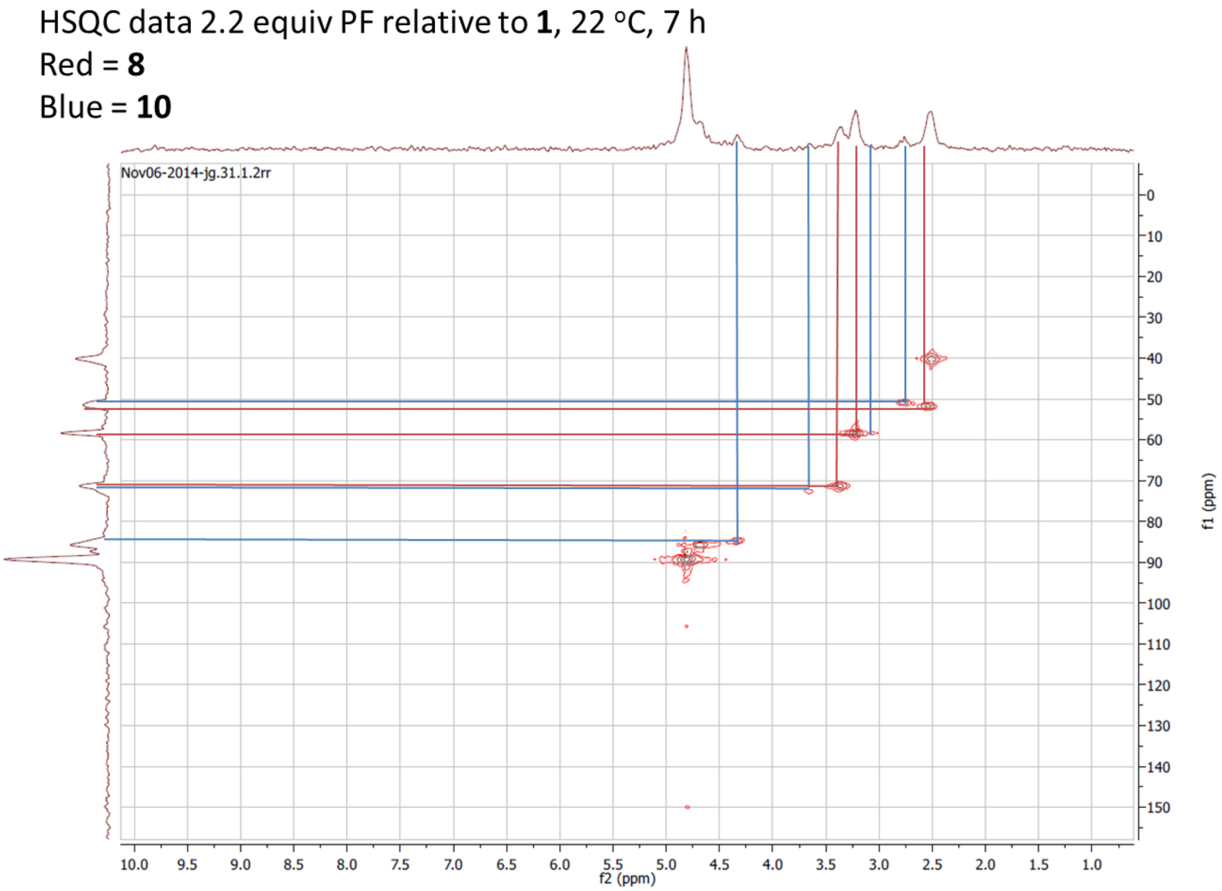

Figure S10. HSQC of thermodynamic products with stoichiometric excess of paraformaldehyde. HSQC data show correlations between carbon and proton signals corresponding to **10** and **8** after 7 hours reaction of **1** with paraformaldehyde at room temperature in d_6-DMSO.

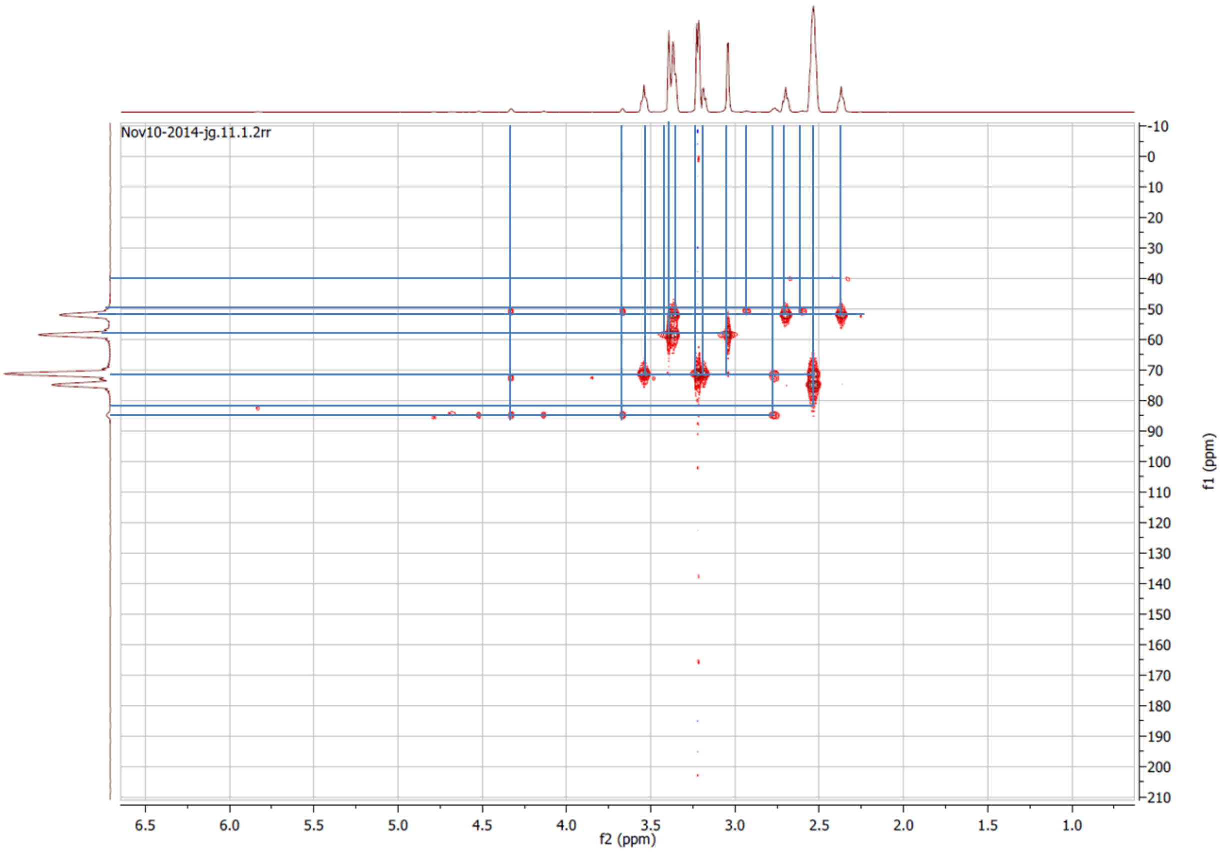

Figure S11. HMBC of thermodynamic products with stoichiometric excess of paraformaldehyde. HMBC data showing through-bond correlations of products formed from reacting **1** with paraformaldehyde at room temperature after 6 hours.

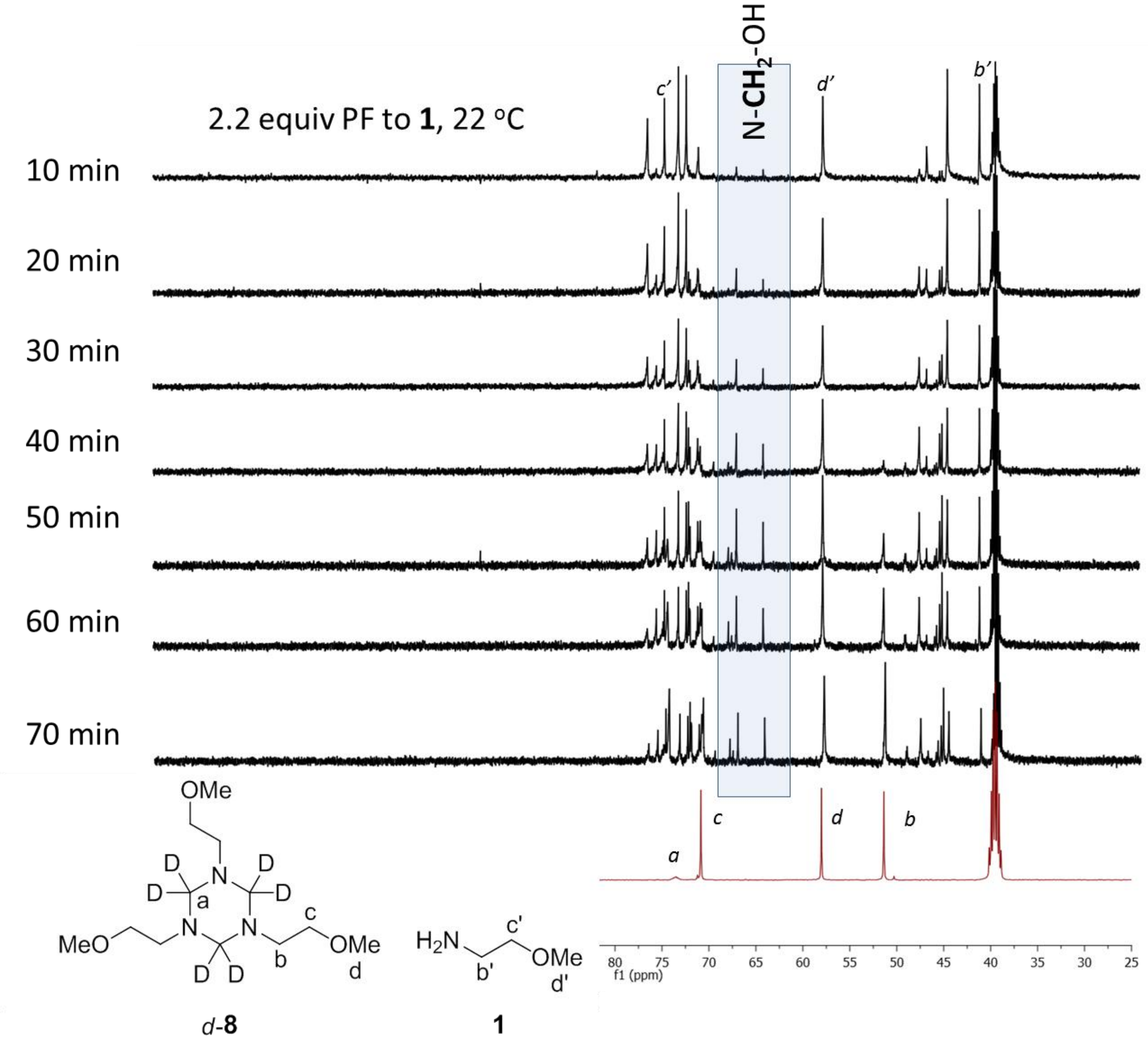

Figure S12. ^{13}C NMR data over first hour with stoichiometric excess of paraformaldehyde. Kinetic ^{13}C NMR data of the first 70 minutes of reaction of **1** with 2.2 equivalents of paraformaldehyde in d_6-DMSO. Hexahydrotriazine is first observed in the 40 minute scan. Signals between 60–70 ppm correspond to hemiaminal species that are formed over the course of the reaction.

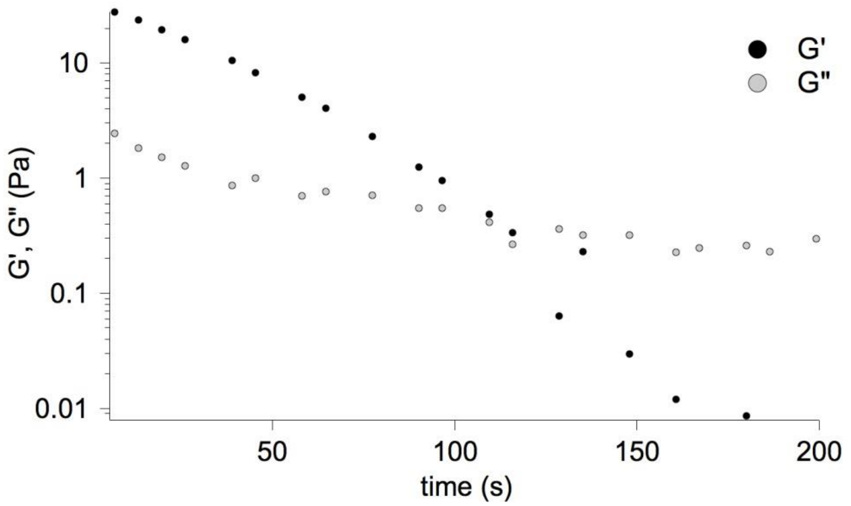

Figure S13. Isothermal mechanical testing of H/H organogel sample. Storage (G’) and Loss (G”) Moduli of a H/H HDCN held at 110 °C for t=200s.

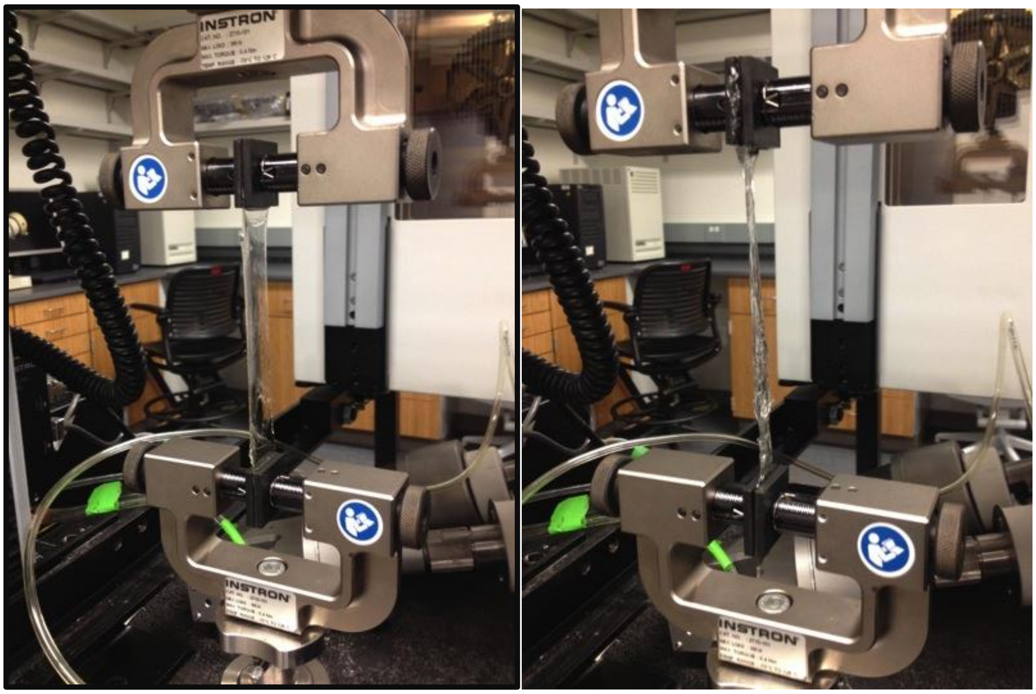

Figure S14. Instron images of H/L and H/H gels. (**A**) H/L HDCN organogel cast from solution stretches to 150% strain in an Instron tensile testing apparatus. (**B**) H/H HDCN

organogel cast from solution stretches to 200% strain in an Instron tensile testing apparatus without loss of solvent.

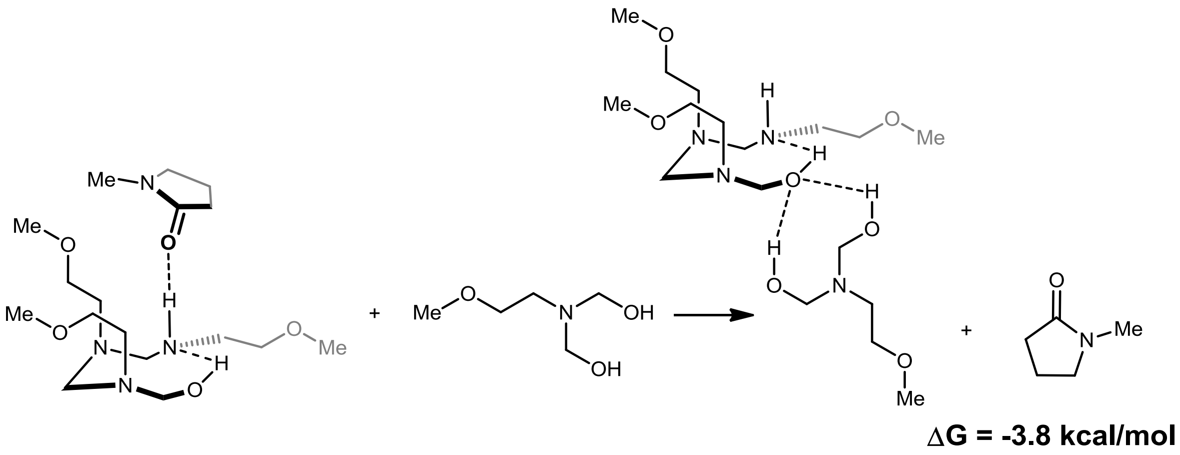

Figure S15. DFT calculations for bishemiaminal H-bonding. DFT calculations show that bishemiaminal **3** can displace NMP and is exergonic by ~4 kcal/mol.

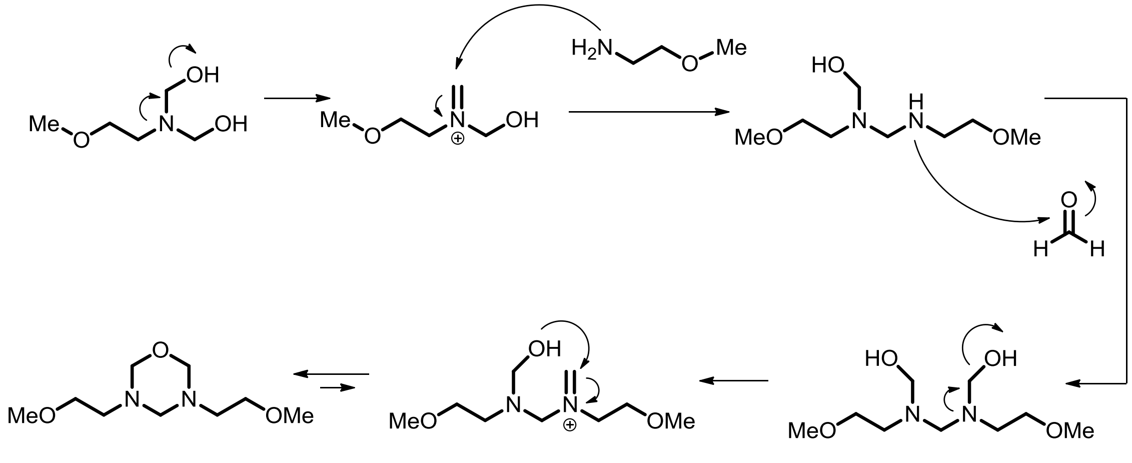

Figure S16. Production of oxadiazinane from bishemiaminal. Plausible arrow-pushing mechanism for the formation of **10** from **3**.

Supplementary Tables

Table S1. Amounts of PEG-diamine and paraformaldehyde used to synthesize HDCNs for mechanical testing.

HDCN (Temp/PF)	Mass PEG-diamine (g)	PEG-diamine (mmol)	PF Equiv. per PEG	PF (mmol)	mass PF (g)
L/L	0.400	0.087	2.5	0.220	0.0065
L/H	0.400	0.087	2.5	0.220	0.0065
H/L	0.400	0.087	4.4	0.380	0.0115
H/H	0.400	0.087	4.4	0.380	0.0115

A series of complementary NMR studies were conducted using aliquots from HDCN-forming reactions prior to the gelation time (t=40 min.) at 50 °C. Aliquots were taken from the same sample at predetermined time intervals and analyzed by ^{1}H-NMR (d_6-DMSO). The reaction conditions for synthesizing HDCNs for ^{1}H-NMR studies are shown below (Table S2).

Table S2. Amounts of PEG-diamine and paraformaldehyde used to synthesize HDCNs for characterization by ^{1}H-NMR. The conditions for each reaction are associated with specific figures presented in the Supplemental Information.

Figure ID	Mass PEG-diamine (g)	mmol PEG-diamine	PF Equiv./PEG	mmol PF	mass PF (g)
S2	0.400	0.087	4.4	0.383	0.011
S4	0.400	0.087	4.4	0.383	0.011

NMR studies conducted using 2-methoxethylamine, a compound electronically and structurally similar to the end of a PEG-diamine molecule, were carried out in d_6-DMSO unless otherwise noted. The reaction conditions used for model experiments are shown in Table S3.

Table S3. Amounts of 2-methoxyethylamine and paraformaldehyde used to study the different products formed in HDCN reactions by ^{1}H-NMR and ^{13}C-NMR. The conditions for each reaction are associated with specific figures presented below.

Figure ID	Mass 2-MEA (g)	mmol	Molar equiv. PF	mmol PF	mass PF (g)	Reaction Solvent	NMR Solvent
S3	0.020	0.266	2.2	0.586	0.018	d_6-DMSO	D_2O

Supplementary Discussion

Figure S3 indicates that hexahydrotriazines formed in the model reaction at higher temperatures do not break down in the presence of D_2O within 48 h. Thus, networks containing the cyclized hexahydrotriazine repeating unit are not hydrolyzable. However, the network formed by PEG diamine and paraformaldehyde completely reverts back to the starting material (PEG-diamine) in the presence of water, as indicated in Figure S4 and Figure S5.

A kinetic study of the products of the reaction of methoxyethylamine (**1**) with paraformaldehyde in d_6-DMSO in Figure S6 illustrates that reactive hemiaminal intermediates are produced and consumed over the course of an hour. A broad singlet at δ = 3.27 ppm appears at the end of reaction, which we have attributed to the formation of hexahydrotriazine (**8**). Additionally, we see the emergence of hemiaminals and bis-hemiaminals (**2** and **3**) in the δ = 3.40–3.70 ppm range.

HDCN-forming reactions require a higher temperature to proceed, but model compound kinetic analyses were carried out at ambient temperatures in order to detect transient species that form over the course of the reaction time for HDCN reactions. Models of the molecular-level formation of HDCN crosslink junctions highlight that the observed mechanical properties are governed by the chemistry of network formation.

Self-healing and temperature-dependent mechanical properties observed in HDCNs correspond to molecular-level network defects which gives rise to self-healing elastomer networks.

The model compound is capable of forming hexahydrotriazine linkages, whereas network topological constraints prevent PEG-diamine from forming hemiaminal cross-link junctions. We attribute this to the constraint imposed on diamines at cross-link junctions. As the reaction proceeds, there are less free diamine end-groups, and even fewer mobile difunctional PEG-diamine chains. PEG-diamine end-groups are tethered to a cross-link junction, limiting the local mobility of free amines in the network. Thus, the formation of hexahydrotriazine cross-link junctions in HDCNs is hindered by topological constraints in the network resulting from the formation of hemiaminal linkers. An HDCN organogel was soaked in H_2O overnight and lyophilized. The gel was insoluble in NMP after lyophilization, which we attribute to the formation of hexahydrotriazine linkages upon removal of NMP under vacuum.

Additionally, a portion of a HDCN network was degraded in H_2O under an active stream of argon and then subsequently analyzed using ^{1}H-NMR (Figures S4 and S5). When the spectrum for a degraded HDCN was compared to a spectrum taken during the HDCN forming reaction at $t < t_{gel}$ (~30 min.), the NMR spectrum for the degraded HDCN indicates that we have reverted the network to primarily PEG diamine. The triplet at δ = 3.13 ppm and singlet at δ = 2.87 ppm, attributable to H-bonded NMP in the network, disappears after water treatment. Powdered PEG diamine can be recovered after the HDCN structure has been reverted in water.

The reaction mechanism proposed in Figure 2 relies upon the presence of water. We argue that the concentration of water required to drive the formation of hemiaminal linkages is low (3 mg to have a stoichiometric equivalency). The spectra shown in Figure S7 indicate that dry conditions drastically slow the reaction. The kinetics are significantly slowed compared to “wet” conditions and mono-hemiaminal **2** is cleanly formed within the first 5 min at room temperature. After 1 hour, two more hemiaminal species are formed likely corresponding to **3** and other hemiaminal intermediates upon the condensation pathway to **8**. After 18 hours in anhydrous conditions with stoichiometric paraformaldehyde, only **8** is observed, compared to after 1 hour with molecular sieves where a mixture of starting material and hemiaminals are the only products (Figure S8). This is in agreement with a recently reported computational and experimental study on a similar system.[1]

Figure S13 presents the decrease in the storage modulus of H/H HDCNs over a 200s equilibration at 110°C. After heating at 110°C for 200 s, the storage modulus crosses over the loss modulus and the liquid-like behavior dominates, indicating that the materials are thermally reprocessable and can be reverted back to liquid precursors at higher temperatures. We attribute this reprocessability to the reversion of HDCN trimer linkages to monohemiaminals at higher temperatures. Monohemiaminals revert to form hemiaminal trimer linkages upon cooling.

Supplementary Methods

^{1}H NMR Characterization of HDCN Networks and Model Compounds

We studied the time-dependent evolution of a PEG-HDCN network by sampling aliquots of HDCN precursors in a 50°C network-forming reaction. The signal for

hexahydrotriazines as well as hemiaminals in the PEG-diamine HDCN forming reaction, if present, would be engulfed by the signal for PEG (~3.30 ppm). The signals for hexahydrotriazine (**8**) and hemiaminals (**6** & **7**) are not observed in Figure S2.

The model compound for PEG-diamine (2-methoxyethylamine, **1**) was reacted with 2.2 equiv. paraformaldehyde at 110°C for 1.5 hours in d_6-DMSO, forming hexahydrotriazine oligomers (broad singlet at δ ~ 3.27 ppm). D_2O was added to the NMR tube and allowed to react for ~18 h at room temperature. The unchanged hexahydrotriazine signal at δ ~ 3.27 ppm after exposure to D_2O indicates that the hexahydrotriazine linkages did not revert overnight. However, the PEG HDCN reverts to PEG diamine after standing in D_2O, substantiating kinetically trapped hemiaminal products and not the production of HT. Computational experiments also support the stability of HTs in water (Figure S6).[1]

Computational Methodology

All calculations were performed with the GAMESS-US[2–4] suite of packages using the dispersion-corrected5 B3LYP[6–9] (B3LYP-D3) density functional theory (DFT) method. Geometry optimizations were performed with the 6-311+G(2d,p)[10] basis set followed by single point energy calculations with the aug-cc-pVTZ[11,12] basis set. A continuum dielectric with the IEF-cPCM[13–15] method was utilized to represent reaction conditions and all reported stationary points were optimized in implicit DMSO solvent at 383 K. Reported energies are free energies in kcal/mol. Only vibrational free energy corrections to the electronic energy at 383 K were used in accordance with recommendations for molecules optimized in implicit solvent.[16] Normal modes of all

structures were examined to verify that equilibrium structures possess no imaginary frequencies and that one imaginary frequency corresponding to bond formation or bond breaking was obtained for transition state structures. Intrinsic reaction coordinate (IRC) calculations were also performed to verify that transition states are connected to reactant complexes and intermediates on the potential energy surfaces of reactions. Partial atomic charges have been computed from an electrostatic potential fit that preserves total molecular charge, dipole and quadrupole moments.

The following settings were used in GAMESS-US.

$dft   nrad=96 nleb=590 $end

$tescav ntsall=240 $end

$force temp=383 $end

Gradient tolerance [a.u.]: 0.00003 for minima and saddle points

Mechanical Testing of HDCNs

Dynamic Mechanical Characterization

The dynamic properties of the HDCN organogels were characterized using a TA Instruments AR2000 rheometer equipped with a 25mm parallel plate geometry. A Peltier plate system was used to control the reaction temperature. A solvent trap was used for all measurements to minimize the loss of NMP at elevated temperatures. Temperature-dependent oscillatory shear rheology experiments were used to demonstrate the thermally healable nature of dynamic covalent networks. Gels with high and low paraformaldehyde content were immediately placed on a preheated plate

(110°C) after the reaction was completed. The storage and loss moduli were measured (ω = 1Hz, 1% strain) while cooling the gel from 110°C °C to 25°C at a rate of 4.25°C/min. Gels were immediately reheated from 25°C to 110°C. A 1% strain value deformation was well within the linear viscoelastic regime for this material.

Compressive Mechanical Characterization

The compressive modulus and hysteresis behavior of HDCN organogels were characterized using an Instron 5800 testing station equipped with a 10 kN load cell. The hysteresis behavior of gels was explored by cyclically loading and unloading HDCN gels within the elastomeric range of organogels. The stress-strain behavior of these gels was characterized using compressive mechanical analysis. Samples for compressive mechanical testing were formed in 25 mm diameter reaction vials. The compressive mechanical testing protocol was developed using this specimen size. Compressive mechanical testing experiments were conducted at ambient conditions; HDCNs were compressed at a rate of 0.1 (mm/mm)/min**.** Hysteretic compressive experiments were conducted within the range of 60% deformation at the same compressive load and unload rate. The energy dissipated by compressing the elastomeric gel networks was quantified by measuring the area between loading and unloading cyclic curves. The energy dissipated is expressed in terms of the Joules per mol PEG to normalize for the polymer content in each sample.

Supplementary References

1. Jones, G. O., García, J. M., Horn, H. W., Hedrick, J. L. Computational and Experimental Studies on the Mechanism of Formation of Poly (hexahydrotriazine) s and Poly (hemiaminal) s from the Reactions of Amines with Formaldehyde. *Org. Lett.* **16**, 5502–5505 (2014).
2. Schmidt, M. W. et al. General atomic and molecular electronic structure system. *J. Comput. Chem.* **14**, 1347–1363 (1993).

3. Gordon, M. & Schmidt, M. in Theory and Applications of Computational Chemistry: The First Forty Years (eds. Dykstra, C., Frenking, G., Kim, K. & Scuseria, G.) 1167–1189 (Elsevier, 2005).
4. Schmidt, M. W. et al. GAMESS 2012 R2. at <http://www.msg.ameslab.gov/GAMESS/GAMESS.html>
5. Grimme, S., Antony, J., Ehrlich, S. & Krieg, H. A consistent and accurate ab initio parametrization of density functional dispersion correction (DFT-D) for the 94 elements H-Pu. *J. Chem. Phys.* **132**, 154104–154119 (2010).
6. Becke, A. D. Density-functional thermochemistry. III. The role of exact exchange. *J. Chem. Phys.* **98**, 5648–5652 (1993).
7. Lee, C., Yang, W. & Parr, R. G. Development of the Colle-Salvetti correlation-energy formula into a functional of the electron density. *Phys. Rev. B* **37**, 785–789 (1988).
8. Vosko, S. H., Wilk, L. & Nusair, M. Accurate spin-dependent electron liquid correlation energies for local spin density calculations: a critical analysis. *Can. J. Phys.* **58**, 1200–1211 (1980).
9. Stephens, P. J., Devlin, F. J., Chabalowski, C. F. & Frisch, M. J. Ab Initio Calculation of Vibrational Absorption and Circular Dichroism Spectra Using Density Functional Force Fields. *J. Phys. Chem.* **98**, 11623–11627 (1994).
10. Krishnan, R., Binkley, J. S., Seeger, R. & Pople, J. A. Self-consistent molecular orbital methods. XX. A basis set for correlated wave functions. *J. Chem. Phys.* **72**, 650–654 (1980).
11. Woon, D. E. & Dunning, T. H. Gaussian basis sets for use in correlated molecular calculations. V. Core-valence basis sets for boron through neon. *J. Chem. Phys.* **103**, 4572–4585 (1995).
12. Kendall, R. A., Dunning, T. H. & Harrison, R. J. Electron affinities of the first-row atoms revisited. Systematic basis sets and wave functions. *J. Chem. Phys.* **96**, 6796–6806 (1992).
13. Barone, V. & Cossi, M. Quantum Calculation of Molecular Energies and Energy Gradients in Solution by a Conductor Solvent Model. *J. Phys. Chem. A* **102**, 1995–2001 (1998).
14. Cossi, M., Rega, N., Scalmani, G. & Barone, V. Energies, structures, and electronic properties of molecules in solution with the C-PCM solvation model. *J. Comput. Chem.* **24**, 669–681 (2003).
15. Miertuš, S., Scrocco, E. & Tomasi, J. Electrostatic interaction of a solute with a continuum. A direct utilization of AB initio molecular potentials for the prevision of solvent effects. *Chem. Phys.* **55**, 117–129 (1981).
16. Ribeiro, R. F., Marenich, A. V., Cramer, C. J. & Truhlar, D. G. Use of Solution-Phase Vibrational Frequencies in Continuum Models for the Free Energy of Solvation. *J. Phys. Chem. B* **115**, 14556–14562 (2011).
