# Supplementary material for: Supramolecular motifs in dynamic covalent PEG-hemiaminal organogels
Source: Nat Commun. 2015 Jul 15;6:7417. doi: 10.1038/ncomms8417 (PMC4518264; doi:10.1038/ncomms8417)
Supplement: Supplementary Data 1 — Cartesian coordinates and energies for reactants, intermediates and transition structures in the reaction of methylamine with formaldehyde [file ncomms8417-s2.doc]

**Cartesian coordinates and energies for reactants, intermediates and transition structures in the reaction of methylamine with formaldehyde.**

**formaldehyde**

E [6-311+G(2d,p); Hartrees] = -114.5524522739

E [aug-cc-pVTZ; Hartrees] = -114.5588457619

Gvib [kcal/mol] = 16.654

C 1.19934515 -0.00000000 0.00013919

O -0.00828625 0.00000000 0.00018275

H 1.77947055 -0.93920390 -0.00016097

H 1.77947055 0.93920390 -0.00016097

h4o2

E [6-311+G(2d,p); Hartrees] = -152.9461959514

E [aug-cc-pVTZ; Hartrees] = -152.9551522105

Gvib [kcal/mol] = 26.846

O -0.96499714 -0.15290061 0.69729254

H -0.72886145 -0.24288907 1.62995727

H -1.45607140 -0.95789037 0.48627172

O 1.29065398 0.20391168 -0.98316912

H 0.50962618 0.10208269 -0.40494050

H 1.24153758 1.10822222 -1.31466410

**methylamine**

E [6-311+G(2d,p); Hartrees] = -95.9036692990

E [aug-cc-pVTZ; Hartrees] = -95.9097269115

Gvib [kcal/mol] = 40.072

N -0.00008202 -0.14857174 -0.90441256

C 0.00001854 -0.12934515 0.56766692

H 0.81031055 0.35411249 -1.25472723

H -0.88040537 -0.65933849 0.93616229

H -0.00065763 0.87657265 1.00624936

H 0.88105188 -0.65828960 0.93613888

H -0.80938060 0.35591385 -1.25465328

**meint1mono**

E [6-311+G(2d,p); Hartrees] = -363.4307323972

E [aug-cc-pVTZ; Hartrees] = -363.4513692839

Gvib [kcal/mol] = 86.528

N 0.94862734 -0.18251138 0.34925926

H 0.52378930 -0.26176700 -0.59532311

H 1.29657024 -1.10755569 0.60247093

C 2.04771667 0.81470801 0.33649907

O -2.65360104 -0.68962036 -0.64270500

H -2.06484126 -0.78790261 0.20777222

H -3.05907011 -1.55227985 -0.78966311

C -0.23292021 0.13670497 1.34822734

O -1.10915867 -0.85919784 1.36461247

H 0.29381583 0.31944721 2.29985384

H -0.60475551 1.10475661 0.96461634

O -0.45605111 -0.26831690 -2.12555917

H -1.34751703 -0.40547356 -1.70768395

H -0.51199925 0.56286764 -2.61239131

H 2.49538545 0.86868974 1.32660958

H 1.63449104 1.78473576 0.06700082

H 2.79951833 0.52271525 -0.39359621

**meint2mono**

E [6-311+G(2d,p); Hartrees] = -363.4277814471

E [aug-cc-pVTZ; Hartrees] = -363.4481319469

Gvib [kcal/mol] = 86.009

N 0.91372421 -0.19152190 0.31200014

H 0.48113587 -0.25726699 -0.64692362

H 1.27758311 -1.11570883 0.54808042

C 2.00676101 0.81704120 0.30536161

O -2.55847300 -0.60969092 -0.65680364

H -1.80400071 -0.80746932 0.45870324

H -3.00102162 -1.43911244 -0.86989852

C -0.22410084 0.11842871 1.29455267

O -1.12193438 -0.90287623 1.31473555

H 0.25534565 0.25821103 2.26666603

H -0.63646825 1.07378355 0.94821410

O -0.47295828 -0.27599913 -2.05600582

H -1.38638026 -0.40615826 -1.57631060

H -0.52929886 0.55432139 -2.54357233

H 2.45562965 0.87118867 1.29475185

H 1.58675675 1.78370772 0.03534519

H 2.75533815 0.52594208 -0.42745910

**meint3mono**

E [6-311+G(2d,p); Hartrees] = -363.4423948465

E [aug-cc-pVTZ; Hartrees] = -363.4633849608

Gvib [kcal/mol] = 86.768

N 0.87168715 -0.17452591 0.32889646

H 0.13926814 -0.25833708 -1.24845027

H 1.28226228 -1.08095889 0.54249563

C 1.93598010 0.83977568 0.27175244

O -2.70791828 -0.60801331 -0.78095636

H -1.69225018 -0.83123930 0.70307393

H -3.17153441 -1.41584215 -1.03409965

C -0.11794209 0.12293137 1.37486689

O -1.08191299 -0.89587593 1.46914208

H 0.35614973 0.20035112 2.36065126

H -0.57023000 1.09190258 1.13289204

O -0.35855864 -0.32114453 -2.12136350

H -1.96137244 -0.51399114 -1.41943643

H -0.21845076 0.51863707 -2.57555698

H 2.44546838 0.97889141 1.23304226

H 1.50716030 1.79763572 -0.02964019

H 2.67323268 0.54629055 -0.47511335

**meint4bis**

E [6-311+G(2d,p); Hartrees] = -478.0073553180

E [aug-cc-pVTZ; Hartrees] = -478.0342517980

Gvib [kcal/mol] = 106.359

N -0.24439719 0.26866065 -0.47139094

H -0.41058365 0.23110315 -1.49753093

C 0.32752714 -1.05406579 -0.03579344

C 0.66952020 1.39807730 -0.17220435

O -3.48404491 -0.32432879 -2.38578694

H -3.14735057 -0.45997158 -1.43135791

H -3.79296957 -1.18768487 -2.68524225

C -1.66606632 0.48313173 0.18892716

O -2.47509012 -0.56297023 -0.02149572

H -1.41482035 0.66609104 1.24637905

H -1.99954287 1.43110157 -0.26439004

O -0.98978052 0.32182564 -3.21148218

H -1.94318966 0.10033080 -3.05540524

H -0.98392634 1.18116496 -3.65063536

H 0.86462076 1.43101178 0.89833270

H 0.19744739 2.32695919 -0.48539298

H 1.60453805 1.26312095 -0.71258953

H 0.34421166 -1.03177888 1.05984106

H 1.34654357 -1.12901271 -0.41512832

O -0.43506244 -2.09717000 -0.54995552

H -1.37104484 -1.84027875 -0.33307595

**meint4mono**

E [6-311+G(2d,p); Hartrees] = -363.4338917559

E [aug-cc-pVTZ; Hartrees] = -363.4550646304

Gvib [kcal/mol] = 85.052

N 1.08881466 -0.13129294 0.33868700

C 2.23885608 0.77716933 0.35900143

O -2.61054734 -0.82092437 -0.64387215

H -2.04978348 -0.94570779 0.15329645

H -2.98907768 -1.68773499 -0.83594802

C 0.08547669 0.20649923 1.31989104

O -0.84535461 -0.87219742 1.48474324

H 0.58309475 0.42226611 2.27048871

H -0.53742668 1.06883014 1.04963679

O -0.56152770 -0.02377279 -2.35126470

H -1.36034570 -0.28370988 -1.84413094

H -0.70302883 0.89806789 -2.59737473

H 2.75798743 0.69598421 1.31683154

H 1.96949685 1.83221691 0.20617598

H 2.93603895 0.48818403 -0.42806779

H -0.32842044 -1.68398179 1.59170435

H 0.66496654 -0.12061289 -0.58835944

**meint5bis**

E [6-311+G(2d,p); Hartrees] = -478.0162751491

E [aug-cc-pVTZ; Hartrees] = -478.0434755222

Gvib [kcal/mol] = 106.012

N 0.19036572 0.25920756 -0.40469063

H -0.56001523 0.18696126 -2.02145931

C 0.84128666 -1.02610701 -0.11220610

C 1.18204506 1.34487266 -0.47036845

O -3.43059636 0.08003001 -1.53230689

H -2.47099286 -0.26081850 -0.08227876

H -3.98264138 -0.66494568 -1.80083558

C -0.83544742 0.58145165 0.60535127

O -1.84767928 -0.40049550 0.66764704

H -0.38391212 0.62844368 1.60508258

H -1.24834672 1.56343942 0.35803727

O -1.06901901 0.18164903 -2.88258168

H -2.68282498 0.10942500 -2.17560045

H -0.87208900 1.02070844 -3.31700211

H 1.73161432 1.46906636 0.47229947

H 0.67736537 2.28408084 -0.69900374

H 1.89716527 1.13451731 -1.26544707

H 1.10629157 -1.08106371 0.95595326

H 1.75473941 -1.09218107 -0.70200770

O 0.03754908 -2.12981661 -0.48055872

H -0.81042419 -2.01555877 -0.02294632

**meint5mono**

E [6-311+G(2d,p); Hartrees] = -363.4335576045

E [aug-cc-pVTZ; Hartrees] = -363.4544902766

Gvib [kcal/mol] = 80.693

N 1.30547009 0.53466109 0.50962513

C 2.49376304 -0.30459412 0.40420485

O -2.65233326 -1.16565519 -0.51454933

H -1.66075635 -1.22980130 1.06270991

H -2.74259034 -2.02317136 -0.94789220

C 1.35157281 1.58184750 1.21797815

O -1.02375983 -1.13488077 1.79904502

O -0.53332641 0.20523148 -1.59799680

H -1.95336913 -0.68647394 -1.01615974

H -0.72713455 1.08344154 -1.94836127

H 3.33593804 0.07386483 0.99238643

H 2.78420906 -0.36957774 -0.64624434

H 2.24350096 -1.31424397 0.73569730

H -0.31446627 -0.58997895 1.42749868

H 0.09003895 0.34609829 -0.84137465

H 2.24734028 1.88928820 1.76522955

H 0.46876427 2.21206655 1.29590374

**meint6bis**

E [6-311+G(2d,p); Hartrees] = -478.0138830990

E [aug-cc-pVTZ; Hartrees] = -478.0418846090

Gvib [kcal/mol] = 104.352

H -0.05191798 -0.47795185 0.42000948

H -1.65878117 -3.91501916 -2.19540678

O -2.19800250 -0.72522843 -1.95848851

H -2.26609347 -0.68574349 -0.97818293

H -3.10305032 -0.66499184 -2.28827353

C -0.67918592 -0.66583891 1.29075467

O -2.00251450 -0.94618670 0.74487002

N -0.10022960 -1.70746176 2.07088424

H -0.74742905 0.24387227 1.89123268

O -1.06873931 -3.24609921 -1.82688891

H -1.51981767 -2.38562161 -1.97818062

H -2.67558094 -0.73953583 1.40399120

C -0.81918830 -2.02945194 3.30086912

C 0.42839164 -2.84826328 1.36084015

H -1.01427176 -1.11127924 3.85932072

H -0.20103258 -2.68006132 3.92191709

H -1.77213082 -2.53815217 3.11821997

H 1.07399241 -3.39615553 2.04980122

H 1.03415067 -2.47743857 0.52892539

O -0.52790090 -3.79564970 0.88284197

H -0.80984482 -3.53159026 -0.01523462

**meint7bis**

E [6-311+G(2d,p); Hartrees] = -477.9851530557

E [aug-cc-pVTZ; Hartrees] = -478.0118898726

Gvib [kcal/mol] = 101.785

H -0.37601528 -0.22246074 0.54920425

H -2.48675566 -3.63613245 -1.40066883

O -1.27003723 -0.97154347 -1.44298734

H -2.35671167 -0.60787223 -0.38951805

H -1.46860938 -0.57668439 -2.29918999

C -0.61475065 -0.58157171 1.54182147

O -2.95192195 -0.38221568 0.41240256

N -0.26246768 -1.76027993 1.87531716

H -1.11877930 0.05274048 2.25576072

O -1.54513729 -3.43646884 -1.45546390

H -1.45913626 -2.36548067 -1.51175454

H -3.45240640 -1.18343195 0.60451078

C -0.56319639 -2.34524157 3.18629411

C 0.48779788 -2.64937893 0.91704848

H -1.15434231 -1.64764521 3.77251817

H 0.37616851 -2.56518540 3.69325393

H -1.11288773 -3.27061725 3.02991855

H 1.45452182 -2.83182204 1.38949951

H 0.60635581 -2.07209263 -0.00093563

O -0.17400506 -3.84302126 0.72500967

H -0.77124534 -3.74585561 -0.08009951

**mets1mono**

E [6-311+G(2d,p); Hartrees] = -363.4277739362

E [aug-cc-pVTZ; Hartrees] = -363.4481138102

Gvib [kcal/mol] = 85.915

Number of imaginary frequencies: 1 [177.70 cm-1]

N 0.91855715 -0.19114923 0.31283918

H 0.48899549 -0.25743529 -0.64470276

H 1.28304703 -1.11516683 0.54840652

C 2.00957552 0.81926083 0.31007794

O -2.56059192 -0.60862925 -0.64086745

H -1.82328832 -0.80200721 0.43009801

H -3.01155358 -1.43643148 -0.84224093

C -0.22603875 0.11325853 1.29567528

O -1.12143420 -0.90548008 1.30585465

H 0.25562864 0.25189462 2.26783999

H -0.63373496 1.07202091 0.95038861

O -0.47340771 -0.27729086 -2.06772223

H -1.37894616 -0.40451351 -1.58936551

H -0.53170834 0.55199134 -2.55688635

H 2.45745488 0.87056196 1.30008842

H 1.58778285 1.78619437 0.04367475

H 2.75966240 0.53292118 -0.42315811

**mets2mono**

E [6-311+G(2d,p); Hartrees] = -363.4269571182

E [aug-cc-pVTZ; Hartrees] = -363.4472354046

Gvib [kcal/mol] = 85.319

Number of imaginary frequencies: 1 [735.30 cm-1]

N 0.88773460 -0.18933350 0.29501499

H 0.41184629 -0.23832681 -0.67893749

H 1.24835593 -1.11833920 0.51555744

C 1.99241472 0.80398142 0.26434544

O -2.52652203 -0.60741390 -0.74386460

H -1.74573646 -0.81809890 0.56019022

H -2.93548181 -1.43655123 -1.01704179

C -0.19747051 0.13761339 1.30455649

O -1.11557171 -0.88304779 1.37099049

H 0.29285474 0.26792280 2.27022766

H -0.62405791 1.09077926 0.97612565

O -0.46325430 -0.27485146 -1.96899612

H -1.50509516 -0.42967153 -1.45235421

H -0.48792871 0.56006136 -2.45066026

H 2.47956896 0.84590490 1.23655413

H 1.57772772 1.77906721 0.01578988

H 2.71061565 0.51030398 -0.49749792

**mets3bis**

E [6-311+G(2d,p); Hartrees] = -477.9879087281

E [aug-cc-pVTZ; Hartrees] = -478.0154332625

Gvib [kcal/mol] = 99.481

Number of imaginary frequencies: 1 [36.07 cm-1]

N 0.05293602 0.03279806 -0.38029145

C 0.49147375 -1.24152190 0.15045029

C 1.04938079 1.08817674 -0.20399171

H 0.75594901 -1.09542975 1.20650385

H 1.37320364 -1.65366480 -0.36352494

O -0.52270322 -2.22482139 0.00656456

H -1.36648508 -1.82177815 0.27207023

H -0.16593549 -0.07295763 -1.36996554

O -3.58977308 -0.55886957 -2.41379425

H -3.52477425 -0.75728991 -1.46035299

H -3.99315926 -1.34053786 -2.81180715

C -3.27217574 -0.02715179 1.14708882

O -3.11888858 -0.93686663 0.36090852

H -2.72484016 -0.00094211 2.10053275

H -3.96359153 0.80192232 0.93549309

O -1.00943116 -0.01513079 -3.37898177

H -1.92218997 -0.19109043 -3.06789783

H -1.01715650 0.90149460 -3.67907386

H 1.19479976 1.28797229 0.86105483

H 0.69000572 2.00603283 -0.67122001

H 2.03238139 0.84623186 -0.63753623

**mets3mono**

E [6-311+G(2d,p); Hartrees] = -363.4076332894

E [aug-cc-pVTZ; Hartrees] = -363.4279164582

Gvib [kcal/mol] = 82.037

Number of imaginary frequencies: 1 [458.57 cm-1]

N 0.86622145 0.57976330 0.18557989

C 2.22255970 0.08130160 0.37944382

O -2.04938092 -1.22766840 -0.66819411

H -1.55136823 -1.17962791 0.52762352

H -2.05556253 -2.14650450 -0.95888849

C 0.01962186 0.78155947 1.13525711

O -1.12766350 -1.02291861 1.57888500

H 0.35268539 0.79363013 2.16235791

H -0.94172681 1.20207118 0.88546103

O -0.55766818 0.23657909 -2.16264403

H -1.19840682 -0.36072681 -1.62562375

H -1.08658610 0.96403696 -2.51038174

H 2.49032303 0.13765266 1.43297245

H 2.91479433 0.68567506 -0.20569232

H 2.28517339 -0.95454220 0.04215939

H -0.47455962 -1.71736939 1.72975853

H 0.49020450 0.59268746 -0.78173725

**mets4bis**

E [6-311+G(2d,p); Hartrees] = -477.9985815411

E [aug-cc-pVTZ; Hartrees] = -478.0250357109

Gvib [kcal/mol] = 104.668

Number of imaginary frequencies: 1 [866.77 cm-1]

N -0.54367851 -0.00137835 -0.26448041

C 0.09742789 -1.27566621 0.21633223

C 0.33824236 1.16949511 -0.01627705

H 0.04697262 -1.26725672 1.30984023

H 1.13763189 -1.25968431 -0.10258079

O -0.50556108 -2.39813003 -0.34609914

H -1.44388260 -2.37696420 -0.09129673

H -0.72057888 -0.06786953 -1.34300548

O -3.46798933 -0.61875354 -2.22476996

H -3.11748651 -0.82051215 -0.77913615

H -3.72210267 -1.45963704 -2.62200935

C -1.90783167 0.21641477 0.37714311

O -2.74069441 -0.86794524 0.18769837

H -1.73095007 0.37274686 1.44288301

H -2.29826348 1.13425529 -0.07145113

O -1.17770329 -0.09480999 -2.80514954

H -2.32515214 -0.35077788 -2.60425382

H -1.13264321 0.76351818 -3.24238061

H 0.55514740 1.25254987 1.04809059

H -0.16635057 2.07088626 -0.35763328

H 1.26384171 1.03954113 -0.57269510

**mets5bis**

E [6-311+G(2d,p); Hartrees] = -477.9839157791

E [aug-cc-pVTZ; Hartrees] = -478.0107073248

Gvib [kcal/mol] = 101.120

Number of imaginary frequencies: 1 [319.29 cm-1]

H -0.26346888 -0.18250343 0.59145547

H -2.36912088 -3.76343892 -1.58074759

O -1.53035979 -0.89793003 -1.61736795

H -2.16064312 -0.69197502 -0.48923490

H -2.05819528 -0.54540526 -2.34254324

C -0.64379674 -0.56276647 1.52746408

O -2.58134539 -0.51824642 0.54362150

N -0.28362328 -1.74167717 1.91283063

H -1.07739937 0.11689254 2.24306169

O -1.45974666 -3.44219467 -1.56412860

H -1.51917632 -2.41369747 -1.65230965

H -3.04958527 -1.32179470 0.80268076

C -0.67669862 -2.29786095 3.20984724

C 0.47486806 -2.65820049 1.01735208

H -1.22180795 -1.54962302 3.77932923

H 0.21887092 -2.59806210 3.75467349

H -1.30477222 -3.17208215 3.04737934

H 1.39464687 -2.91019673 1.54819089

H 0.71117434 -2.09481068 0.11313681

O -0.22962866 -3.82403567 0.75542923

H -0.75307907 -3.70455867 -0.08507294
